# Lymphoid Aggregates in the CNS of Progressive Multiple Sclerosis Patients Lack Regulatory T Cells

**DOI:** 10.3389/fimmu.2019.03090

**Published:** 2020-01-15

**Authors:** Luisa Bell, Alexander Lenhart, Andreas Rosenwald, Camelia M. Monoranu, Friederike Berberich-Siebelt

**Affiliations:** ^1^Institute of Pathology, Julius-Maximilian University of Wuerzburg, Wuerzburg, Germany; ^2^Comprehensive Cancer Center Mainfranken, Julius-Maximilian University of Wuerzburg, Wuerzburg, Germany; ^3^Department of Neuropathology, Institute of Pathology, Julius-Maximilian University of Wuerzburg, Wuerzburg, Germany

**Keywords:** ectopic lymphoid follicle, lymphoid aggregate, T-follicular regulatory cell, meningeal inflammation, NFATc1, progressive multiple sclerosis

## Abstract

In gray matter pathology of multiple sclerosis, neurodegeneration associates with a high degree of meningeal inflammatory activity. Importantly, ectopic lymphoid follicles (eLFs) were identified at the inflamed meninges of patients with progressive multiple sclerosis. Besides T lymphocytes, they comprise B cells and might elicit germinal center (GC)-like reactions. GC reactions are controlled by FOXP3^+^ T-follicular regulatory cells (T_FR_), but it is unknown if they participate in autoantibody production in eLFs. Receiving human post-mortem material, gathered from autopsies of progressive multiple sclerosis patients, indeed, distinct inflammatory infiltrates enriched with B cells could be detected in perivascular areas and deep sulci. CD35^+^ cells, parafollicular CD138^+^ plasma cells, and abundant expression of the homing receptor for GCs, CXCR5, on lymphocytes defined some of them as eLFs. However, they resembled GCs only in varying extent, as T cells did not express PD-1, only few cells were positive for the key transcriptional regulator BCL-6 and ongoing proliferation, whereas a substantial number of T cells expressed high NFATc1 like GC-follicular T cells. Then again, predominant cytoplasmic NFATc1 and an enrichment with CD3^+^CD27^+^ memory and CD4^+^CD69^+^ tissue-resident cells implied a chronic state, very much in line with PD-1 and BCL-6 downregulation. Intriguingly, FOXP3^+^ cells were almost absent in the whole brain sections and CD3^+^FOXP3^+^ T_FR_s were never found in the lymphoid aggregates. This also points to less controlled humoral immune responses in those lymphoid aggregates possibly enabling the occurrence of CNS-specific autoantibodies in multiple sclerosis patients.

## Introduction

In multiple sclerosis (MS), not only lymphocytes, but also antibodies (Ab) might attack the myelin-surrounded axons (1). Further evidence for autoaggressive antibodies in MS stems from animal models (2, 3). Intrathecal oligoclonal immunoglobulin bands (OCB), detected by separation of CSF proteins and not present in the corresponding serum, reflect a local B-cell response and indicate the presence of B-cell clonal expansion in the CNS accompanying CNS inflammation. This defines the CNS as a site of ongoing immune reactions and an environment fostering proliferation and survival of B cells and plasma cells (PC), eventually becoming a niche for long-lived PCs (LLPC) (4). The antigen specificity of the OCBs is mostly unknown, although intrathecal Abs were shown to react with self-epitopes (5).

Besides intrathecal OCBs, elevated CSF levels of the chemokine CXCL13 have an especially high prognostic value for a conversion from clinically isolated syndrome to definite MS (6, 7). CXCL13 elicits its effects by interacting with the chemokine receptor CXCR5 and is a selective chemoattractant for lymphocytes forming germinal centers (GC) within follicles of secondary lymphoid organs (SLOs). GCs harbor important steps of T cell-dependent B-cell activation, i.e., affinity maturation through somatic hypermutation (SMH) and class-switch recombination (CSR), which leads to the generation of LLPCs and memory B cells. Augmented, unrestrained GC reactions can lead to severe autoimmune disorders, in which auto-Abs attack various tissues. The GC reaction is conducted by highly specialized CD4^+^ T lymphocytes called T-follicular helper (T_FH_) cells (8, 9). They provide cognate help to GC-B cells, which compete for T_FH_ help by increased affinity for antigen. Like GC-B cells, T_FH_ cells depend on the expression of the chemokine receptor CXCR5, to facilitate repositioning from T-cell zones into B-cell follicles, directly promoting GC-immune responses (10, 11).

In healthy individuals, the GC reaction is a precisely controlled process and involves various regulatory cell types. Notably, impaired function of thymus-derived natural FOXP3^+^ T cells (Treg) escalates GC responses (12). Accordingly, a special subset of Tregs was identified in GCs, which shares characteristics with T_FH_ cells and was named T-follicular regulatory cells (T_FR_s) (13–15). Similar to T_FH_, T_FR_s express CXCR5, ICOS, and PD-1, but in addition, they exhibit typical Treg markers, such as FOXP3, CD25, GITR, and CTLA-4. Both, T_FR_s and T_FH_s express the lineage-specific transcriptional regulator BCL-6 and especially high levels of NFATc1 (16, 17). In T_FR_s, as we could show, NFATc1 facilitates homing by upregulating CXCR5 (17). T_FR_s limit the magnitude of the GC reaction, i.e., the number of GC-B cells and the quantity and quality of secreted immunoglobulins, by direct repression of B cells (15, 18–20). Here, they suppress CSR, SHM and antibody secretion through altering their metabolism, but also inhibit IL-21 and IL-4 secretion in T_FH_ cells. Moreover, T_FR_ cell-mediated CTLA-4-dependent regulation of CD80 /CD86 expression on GC-B cells restrains the number of T_FH_ cells and *vice versa* the GC-reaction (21, 22). Exploring T_FR_s in autoimmune diseases, blood-circulating T_FR_s are reported to be lost in favor of a dramatic increase in T_FH_s and IL-21 levels in systemic lupus erythematosus patients and Sjögren syndrome, which could be connected to disease activity (23, 24). In MS patients, a high T_FH_/T_FR_ ratio in blood also correlates with more severe disease course and—intriguingly—with intrathecal IgG synthesis (25–27).

The finding that CXCL13 is dominantly present in CSF of MS patients suggests an involvement of tertiary lymphoid structures /ectopic lymphoid follicles (eLFs), eliciting GC-like reactions. Those eLFs are generated at sites of chronic inflammation and sustain immunopathological processes (28–30). Indeed, sections from post-mortem brains and spinal cords of secondary progressive MS (SPMS) patients led to the identification of eLFs with B, T, plasma cells, and a network of FDCs producing CXCL13, although they were not described in relapsing-remitting MS (RRMS) and only in a lesser defined state in primary progressive MS (PPMS) (31–34). eLFs were recognized in close apposition with cortical subpial lesions in deep cerebral sulci. Their occurrence associates with a poor clinical disease course and could account for cognitive deficits observed in progressive MS patients. Furthermore, meningeal aggregates and parenchymal infiltrates share related antigen-experienced B-cell clones suggesting B-cell trafficking from eLFs to CNS tissue (35).

However, it is not clear, to what extent eLFs in the CNS of progressive MS patients resemble a GC reaction in SLOs and especially, to what extent they are regulated (36, 37). Therefore, the aim of this study was to evaluate if T_FR_ cells are present and we characterized the subtypes of immune cells in lymphoid aggregates. Serial sections of post-mortem brain and spinal cord samples of SPMS and PPMS patients were triple-stained for specific markers. Follicle-like lymphoid aggregates were repetitively detected, but resembled GCs or at least eLFs only in varying extent, best matching an eLF in a memory state. For sure, CD3^+^FOXP3^+^ Tregs were never discovered in those aggregates, hinting to unleashed GC-like immune responses in the CNS of progressive MS patients.

## Materials and Methods

### Demographic and Clinical Data

This study was performed on a new cohort of autopsy brain and spinal cord tissue from 11 cases with PPMS (5 female, 6 male), 22 with SPMS (19 female, 3 male), two Parkinson‘s disease (PD) cases (1 female, 1 male) and 13 healthy control (HC, 1 female, 11 male) cases obtained from UK Multiple Sclerosis Tissue Bank at Imperial College, London, UK (www.imperial.ac.uk/medicine/multiple-sclerosis-and-parkinsons-tissue-bank) (Supplementary Table 1). All procedures used by the Tissue Bank in the procurement, storage and distribution of tissue have been approved by the relevant National Multicentre Research Ethics Committee (08/MRE09/31), UK, and all tissues supplied are obtained via a prospective donor scheme. Both the donor and next of kin have given informed consent for the use of the donor's brain and spinal cord material for MS research. According to the common procedure, we analyzed sex, age of death, age of disease onset, disease duration, brain weight, CSF pH and death-to-tissue interval of PPMS, SPMS and control cases. We found an earlier death of MS patients in comparison to control cases, a strong difference in gender ratio tending toward more female patients suffering from SPMS than PPMS, and a loss in brain weight in SPMS patients compared to PPMS (Supplementary Table 2).

### Tissue and Lesion Classification

Tissues were pre-characterized by UK Multiple Sclerosis Tissue Bank, treated and kept with high quality (38), allowing scanning of the meninges and gray matter (GM) in brain and spinal cord. Per case, 10 sections of one to four paraffin blocks were obtained, pre-defined into normal-appearing white matter (NAWM), normal-appearing spinal cord (NASC), chronic active lesion (CAL), and chronic lesional spinal cord (CLSC) by UK Multiple Sclerosis Tissue Bank (Table 1). Lesional state was verified with Luxol Fast Blue (LFB) staining for demyelination and immunostaining of CD68, detecting microglia and infiltrating macrophages. Chronic active lesions CAL were defined by minor inflammation in the center of the plaque with some CD68^+^ macrophages, whereas chronic inactive lesions exhibited a silent lesion center with few or no CD68^+^ cells (Supplementary Figures 1A–F).

**Table 1:** Tissue characterization, infiltration and follicle status in brain and spinal cord of progressive multiple sclerosis, Parkinson's disease, and healthy control cases.

**Case/Disease subtype**	**Tissue type**	**Number of infiltrated regions**		Median score of superficial infiltration^*^		Median score of parenchymal infiltration^*^		Total median score of infiltration^*^	Follicle status^**^	
		**Surface**	**Parenchyma**	**Meninges**	**Sulcus**	**Cortex of brain/WM of SC**	**WM of brain/GM of SC**	**All regions**	**Follicle presence**	**Number of follicles in infiltrates**
MS136/SPMS	NAWM	0	0	–	–	–	–	–	–	0/0
	CAL	0	5	–	–	–	2.0	2.0	+	2/5
	CALSC	2	0	2.0	–	–	–	2.0	+	1/2
MS166/SPMS	NAWM	0	0	–	–	–	–	–	–	0/0
	CAL		0	–	–	–	–	–	–	0/0
MS169/SPMS	NAWM	0	0	–	–	–	–	–	–	0/0
MS179/SPMS	CAL	4	1	2.5	2.5	–	3.0	3.0	+	2/5
MS180/SPMS	NAWM	0	0	–	–	–	–	–	–	0/0
	CAL	5	1	–	3.0	2.0	–	2.5	+	3/6
	CALSC	6	2	3.0	3.0	–	2.0	2.5	+	4/8
MS186/SPMS	NAWM	0	0	–	–	–	–	–	–	0/0
	CAL	0	2	–	–	2.0	2.0	2.0	–	0/2
	CALSC	1	1	1.0	–	–	1.0	1.0	–	0/2
MS200/SPMS	CAL	0	2	–	–	2.0	1.0	1.5	–	0/2
MS201/PPMS	NAWM	0	0	–	–	–	–	–	–	0/0
	CAL	0	0	–	–	–	–	–	–	0/0
	CALSC	3	1	2.0	1.0	1.0	–	1.0		0/4
MS202/SPMS	CAL	2	4	1.5	–	3.0	2.0	2.0	+	2/6
	CLSC	4	2	1.5	–	–	1.0	1.0	+	1/6
MS207/SPMS	NAWM	0	0	–	–	–	–	–	–	0/0
	CAL	3	1	–	1.0	–	2.0	1.5	+	1/4
MS211/SPMS	NAWM	0	0	–	–	–	–	–	–	0/0
	CAL	0	2	–	–	–	2.0	2.0	+	1/2
MS293/SPMS	CAL	0	1	–	–	–	1.0	1.0	–	0/1
	CALSC	0	0	–	–	–	–	–	–	0/0
MS313/PPMS	NAWM	0	0	–	–	–	–	–	–	0/0
	CALSC	1	2	–	1.0	–	1.5	1.0	–	0/3
MS325/PPMS	NAWM	0	0	–	–	–	–	–	–	0/0
	CAL	0	1	–	–	–	2.0	2.0	–	0/1
	CALSC	0	2	–	–	2.0	1.0	1.5	–	0/2
MS330/SPMS	CAL	0	0	–	–	–	–	–	–	0/0
	CALSC	3	3	2.0	1.5	–	1.0	1.0	–	0/6
MS338/SPMS	NAWM	0	0	–	–	–	–	–	–	0/0
	CAL	2	1	–	3.0	2.0	–	3.0	+	2/3
	CALSC	1	4	–	2.0	–	1.0	1.0	+	1/5
MS340/SPMS	NAWM	1	0	–	–	1.0	–	1.0	–	0/1
	CAL	0	6	–	–	–	1.0	1.0	–	0/6
MS363/PPMS	CAL	0	0	–	–	–	–	–	–	0/0
	CALSC	1	1	–	1.0	–	1.0	1.0	–	0/2
MS383/PPMS	NAWM	0	0	–	–	–	–	–	–	0/0
	CALSC	0	1	–	–	–	1.0	1.0	–	0/1
MS386/PPMS	NAWM	0	0	–	–	–	–	–	–	0/0
	NASC	0	0	–	–	–	–	–	–	0/0
MS389/SPMS	NAWM	0	0	–	–	–	–	–	–	0/0
	CAL	2	17	2.0	1.0	2.5	2.0	2.0	+	4/19
	CALSC	9	7	3.0	3.0	–	3.0	3.0	+	9/16
MS408/SPMS	CAL	1	1	–	3.0	1.0	–	2.0	+	1/2
	CALSC	2	0	1.5	–	–	–	1.5	–	0/2
MS473/PPMS	NAWM	0	0	–	–	–	–	–	–	0/0
	CAL	0	0	–	–	–	–	–	–	0/0
	NASC	0	0	–	–	–	–	–	–	0/0
MS478/SPMS	NAWM	0	0	–	–	–	–	–	–	0/0
	NASC	0	0	–	–	–	–	–	–	0/0
MS485/PPMS	NAWM	0	0	–	–	–	–	–	–	0/0
	CALSC	1	0	1.0	–	–	–	1.0	–	0/1
MS489/SPMS	NAWM	0	0	–	–	–	–	–	–	0/0
	NASC	0	0	–	–	–	–	–	–	0/0
MS492/PPMS	NAWM	0	0	–	–	–	–	–	–	0/0
MS494/	NAWM	0	0	–	–	–	–	–	–	0/0
	CAL	0	2	–	–	2.0	1.0	1.5	–	0/2
	NASC	0	0	–	–	–	–	–	–	0/0
MS497/SPMS	CAL	0	8	–	–	2.5	2.0	2.0	+	2/8
MS500/PPMS	CAL	2	4	2.5	–	–	2.5	2.5	–	0/6
MS503/SPMS	NAWM	0	1	–	–	–	1.0	1.0	–	0/1
	CLSC	0	0	–	–	–	–	–	–	0/0
MS504/SPMS	NAWM	0	0	–	–	–	–	–	–	0/0
MS513/SPMS	CAL	1	7	–	3.0	2.0	2.0	2.0	+	2/8
	NASC	0	0	–	–	–	–	–	–	0/0
C022	NAWM	0	0	–	–	–	–	–	–	0/0
C025	NAWM	0	0	–	–	–	–	–	–	0/0
	NASC	0	0	–	–	–	–	–	–	0/0
C032	NAWM	0	0	–	–	–	–	–	–	0/0
	NASC	0	0	–	–	–	–	–	–	0/0
C036	NAWM	0	0	–	–	–	–	–	–	0/0
	NASC	0	0	–	–	–	–	–	–	0/0
C037	NAWM	0	0	–	–	–	–	–	–	0/0
	NASC	0	0	–	–	–	–	–	–	0/0
C039	NAWM	0	0	–	–	–	–	–	–	0/0
	NASC	0	0	–	–	–	–	–	–	0/0
C045	NAWM	0	0	–	–	–	–	–	–	0/0
	NASC	0	0	–	–	–	–	–	–	0/0
C048	NAWM	0	0	–	–	–	–	–	–	0/0
	NASC	0	0	–	–	–	–	–	–	0/0
C052	NAWM	0	0	–	–	–	–	–	–	0/0
	NASC	0	0	–	–	–	–	–	–	0/0
C054	NAWM	0	0	–	–	–	–	–	–	0/0
	NASC	0	0	–	–	–	–	–	–	0/0
C059	NAWM	0	0	–	–	–	–	–	–	0/0
	NASC	0	0	–	–	–	–	–	–	0/0
C064	NAWM	0	0	–	–	–	–	–	–	0/0
PD032	NAWM	0	0	–	–	–	–	–	–	0/0
	NASC	0	0	–	–	–	–	–	–	0/0
PD034	NAWM	0	0	–	–	–	–	–	–	0/0
	NASC	0	0	–	–	–	–		–	0/0

* *Infiltration score 1, <30 T and/or B cells, but at least five lymphocytes; score 2, 31–60 cells; score 3, >60 mean infiltrated T and B cells per region*.

** *+, region with massive infiltration (score 3) plus positive staining of CD3, CD20, CD35, Ki67, and CD138; –, no or not all criteria are fulfilled*.

*CAL, chronic active lesional brain; CALSC, chronic active lesional spinal cord; NAWM, normal appearing white matter; NASC, normal appearing spinal cord; WM, white matter; GM, gray matter; SPMS, secondary progressive multiple sclerosis; PPMS, primary progressive multiple sclerosis; PD, Parkinson's disease; C, control cases*.

The 5-μm thick formalin-fixated paraffin-embedded (FFPE) sections were entirely screened for infiltration via hematoxylin-eosin staining (H&E) by a blinded neuropathologist identifying regions of infiltration. Modified from (33), the degree of inflammation was evaluated manually counting the number of lymphocytes in each infiltrated region for each MS case (Table 1): score 1, <30 lymphocytes, but at least five = negligible; score 2, 31–60 lymphocytes = moderate; score 3, >60 lymphocytes = abundant. For analysis of the regional occurrence of inflammation, we categorized the infiltrated region into parenchymal infiltration within the cortex in the brain/white matter (WM) of the spinal cord, or WM in the brain/GM in the spinal cord, superficial infiltration within the meninges, or in sulci. These regions of infiltration were annotated and further studied on consecutive sections. Follicle-like structures (F+) were characterized by substantial infiltration (score 3) accompanied by the detection of CD3^+^ T and /or CD20^+^ B cells as well as Ki67^+^ proliferating cells, CD35^+^ or CD21^+^ FDCs, and CD138^+^ plasma cells within the respective region.

### Immunohistochemistry (IHC)

Consecutive, deparaffinized FFPE sections underwent a heat-induced antigen retrieval with Citrate buffer (pH 6.0) and were treated with peroxidase blocking buffer (Dako, #S2023), before incubated with Antibody Diluent for 1 h at room temperature (RT) (Dako, #S3022). Incubation with primary antibodies followed, BCL-6 (1:50, Dako, #M7211), CD21 (1:200, abcam, ab75985), CD35 (1:200, ThermoFisher, #MA5-13122), CD68 (1:200, Clone KiM1P, provided by S.B., Inst. Pathology, Wuerzburg), CXCR5 (1:200, abcam, #ab225575), FOXP3 (1:50, abcam, #ab2034), Ki67 (1:200, ThermoFisher, #14-5698-82) or NFATc1 (1:100, BD Pharmigen, #556602) in Antibody Diluent for 1 h at RT. Then, after washing steps, Histostain-Plus IHC Kit (ThermoFisher, #858943) for FOXP3 and Ki67 or ADVANCE HRP^TM^ (Dako, #K4067, -8, -9) for all other antibodies were used for visualization by avidin-biotin horseradish peroxidase and 3,3′-diaminobenzidine (DAB) as substrate (Dako, #K3468). Sections were viewed with a light microscope (Zeiss Axioskop 2). Images were acquired with a digital camera (Olympus DP26). Positive controls included human tonsils (Supplementary Figures 2A–H). and a follicular lymphoma of the spinal cord (Supplementary Figures 2K–R); negative controls were performed with secondary antibodies only (Supplementary Figures 2I,J,S,T).

### Immunofluorescence (IF)

Consecutive, deparaffinized FFPE sections underwent a heat-induced antigen retrieval with Citrate buffer (pH 6.0) before they were blocked with Antibody Diluent (Dako, #S3022) for 1 h at RT. Sections were incubated with the primary antibodies CD20 (1:200, Dako, #M0755), CD3 (1:100, Dako, #A0452), CD3 (1:50, abcam, #11089), CD4 (1:200, R&D Systems #AF-379-NA), CD8 (1:100, Dako #M7103), CD27 (1:50, Sigma-Aldrich, #HPA038936), CD138 (1:200, BioLegend, # 356502), CD69 (1:100, ThermoFisher, #PA5-84010), CXCR5 (1:200, abcam, #ab225575), FOXP3 (1:100, ThermoFisher, #14-4776-82), NFATc1 (1:100, BD Pharmigen, #556602), and/or PD-1 (1:100, abcam, #ab52587) in Antibody Diluent for 1 h. Secondary antibodies (1:400, all from ThermoFisher)—donkey-anti-goat Alexa Fluor 546 (#A-11056), donkey-anti-mouse Alexa Fluor 647 (#A-31571), donkey-anti-rabbit Alexa Fluor 488 (#A-21206), donkey-anti-rabbit Alexa Fluor 555 (#A-31572), donkey-anti-rat Alexa Fluor 488 (#A-21208), donkey-anti-rat DyLight 550 (#SA5-10027)—were applied in PBS containing 0.05% Tween20 and Hoechst (1:5.000, Sigma, #B2261) for 1 h at RT. After washing with PBS, sections were embedded in Mowiol 4-88 (Roth, #0713). Positive controls included human tonsils (Supplementary Figures 3A,D,G,J,H,P), a follicular lymphoma of the spinal cord (Supplementary Figures 3B,E,H,K,N,Q), and a case of primary CNS lymphoma of the brain (Supplementary Figures 3C,F,I,L,O,R); negative controls were incubated with the secondary antibodies only (Supplementary Figures 3S–D'). For quantitative analysis, images were captured according to the pre-defined infiltrated regions by H&E at 20-x magnification. For representative images, confocal laser-scanning microscopy images were acquired using a Nikon A1R + confocal microscope system equipped with an Eclipse TI-E inverse microscope with coherent sapphire lasers and a visible fiber laser (lines: 405, 488, 561, and 647 nm) (MBP Communications). Plan-Apochromat 60 × NA 1.4 objectives were used for detection in four simultaneous channels. The system was equipped with NIS-Elements Advanced Research Software (Nikon). Brightness was adjusted for each staining according to the control staining using ImageJ 1.6 (National Institute of Health).

### Quantitative Analysis of Cell Subtypes

**Figure 1:**
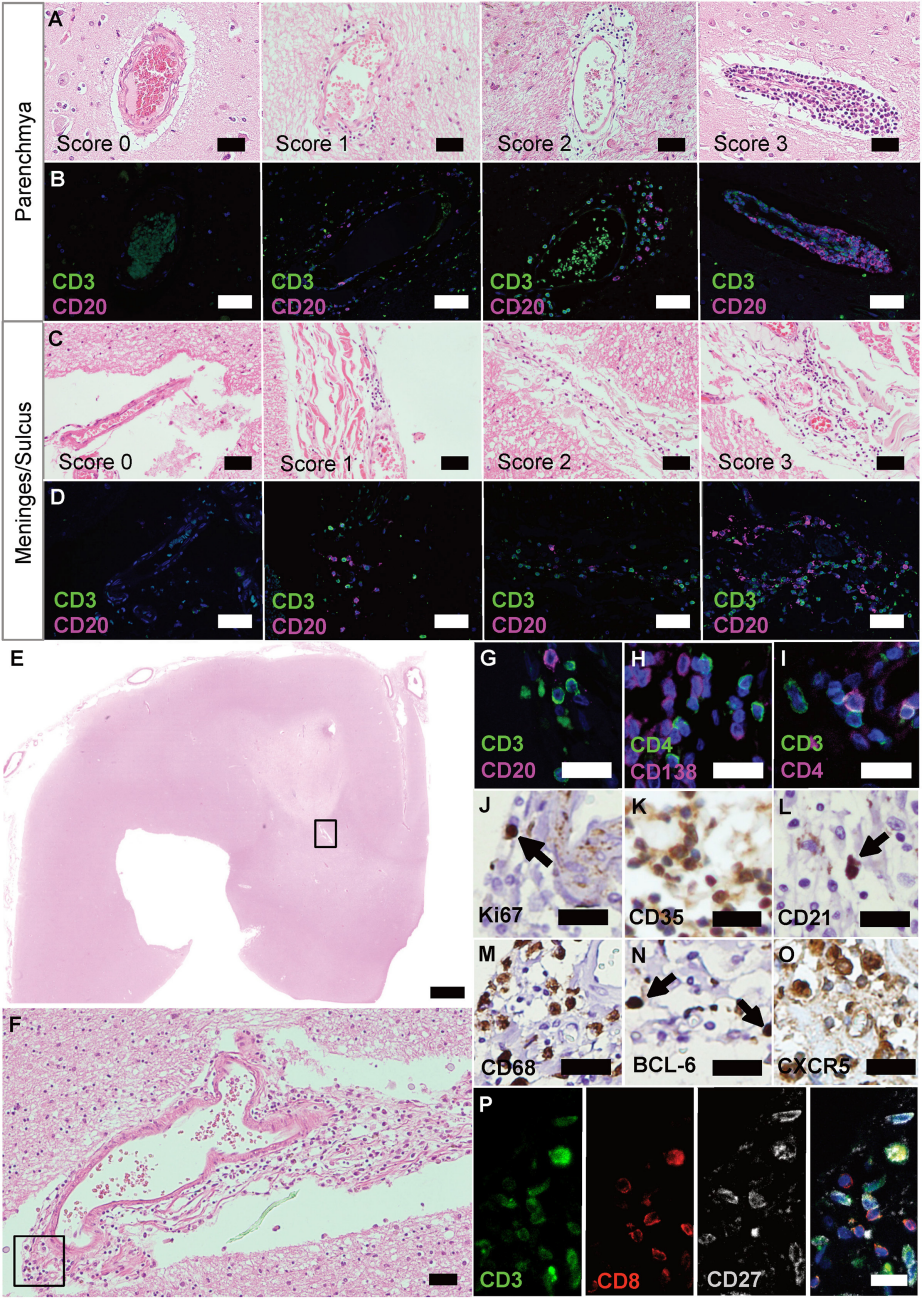
Ectopic lymphoid structures in progressive MS are characterized by infiltration of lymphocytes, FDCs and plasma cells. **(A)** Parenchyma of FFPE sections of brain and spinal cord of progressive MS patients were screened for infiltrated regions by H&E staining. **(B)** IF staining for CD3^+^ T cells and CD20^+^ B cells on serial sections were used to determine the infiltration score. Score 0, no or <5 lymphocytes; score 1, at least five but <30 lymphocytes; score 2, 31 to 60 lymphocytes; score 3, more than 60 lymphocytes. **(C)** Meninges and sulci of FFPE sections of brain and spinal cord of progressive MS patients were screened for infiltrated regions by H&E staining. **(D)** IF staining for CD3^+^ T cells and CD20^+^ B cells on serial sections were used to determine the infiltration score. Score 0, no or <5 lymphocytes; score 1, at least five, but <30 lymphocytes; score 2, 31 to 60 lymphocytes; score 3, more than 60 lymphocytes. **(E)** Whole slides were screened for infiltration on H&E, representative infiltration area depicted in the box **(F)**, and serial sections were stained, depicted in the box **(G–O)**. eLFs are characterized by **(G)** CD3^+^ T cells and CD20^+^ B cells, **(H)** CD4^+^ T cells and CD138^+^ plasma cells, **(I)** CD3^+^ T and CD3^+^CD4^+^ T helper cells **(J)** Ki67^+^ proliferating cells, **(K)** CD35^+^ and, **(L)** CD21^+^ FDCs, **(M)** CD68^+^ macrophages as well as **(N)** BCL-6^+^ and **(O)** CXCR5^+^ GC-like lymphocytes. **(P)** CD3^+^CD8^+^ cytotoxic T cells as well as some CD3^+^CD27^+^ memory T cells were also present in eLFs. Scale bars **(A–D)**, **(F)** indicate 100 μm; **(E)** indicates 2,000 μm; **(G–P)** indicate 50 μm.

Infiltrated regions were identified by microscopically screening of the H&E. If a section contained an infiltrated area, it underwent IHC staining of BCL-6, CD21, CD35, CD68, CXCR5, FOXP3, Ki67, NFATc1 to screen the different cell types within the infiltrates. If the infiltrated area was positive for at least one of these marker, it underwent IF staining and infiltration score was determined. For a representative overview of a whole H&E stained slide (Figure 1E), we used Pannoramic scan II 3D Histech (Sysmex) on 40-x magnification.

IF was carried out on eight consecutive sections: (1) presence of lymphocytes: CD3, CD20; (2) presence of T-helper and plasma cells: CD4, CD138; (3) presence of cytotoxic and memory T cells: CD3, CD8, CD27; (4) presence of Tregs: CD3, FOXP3; (5) presence of T_FH_: CD4, CXCR5; (6) presence of further key features of T_FH_: CD3, CD4, PD-1; (7) presence of NFATc1 in T_FH_: CD4, CXCR5, NFATc1; and (8) presence of presumably tissue-resident T-helper cells: CD4, CD69. We counted the absolute number of cells with each marker within the infiltrated area on IHC as cells per infiltrated mm^2^. To avoid bias, slides were pseudonymized and evaluation was done in a standardized way: After identification of the respective infiltrated region with the help of H&E, single positive cells in all channels were recorded and counted, then the double and triple positive stainings were counted with the help of overlays in ImageJ. By doing so, we determined the number and ratio of CD3^+^ and CD20^+^ lymphocytes, the percentage of CD3^+^FOXP3^+^ Tregs of total CD3^+^ cells within the respective infiltrated region as well as CD4^+^CXCR5^+^ T_FH_ of CD4^+^ cells, and CD4^+^CD69^+^ T_RM_ of CD4^+^ cells. For FOXP3 staining, we additionally used the module cytonuclear (Version v1.6) of the software HALO (Indica Lab) to objectively count the FOXP3^+^ cells within the infiltrated areas *vs*. whole brain section (excluding infiltrated areas).

### Statistical Analysis

Interval-scaled data were presented as mean ± standard deviation (SD), ordinal-scaled data as median ± interquartile range (IQR) or as percentage of categories, and nominal-scaled data as absolute numbers. Statistical analyses were performed using GraphPad Prism5 version 5.0 (GraphPad Software Inc.). Median score of the infiltrates per case was determined based on the absolute number of lymphocytes detected by IF staining. For scoring, IHC (percentage of total cells), and IF (percentage of total cells, CD20/CD3 ratio) data, two-tailed Kruskal-Wallis test with multiple comparisons, or in case of only two groups, the two-tailed, unpaired Mann-Whitney *U* test were used. For the absolute number of infiltrates in F+ vs. F-, we used Fisher's Exact test. For the comparison between the scores in SPMS/PPMS in brain and spinal cord and regions of infiltration, we used Chi-Square test. For demographic and clinical data, we used Kolmogorow–Smirnow test to check for normal distribution followed by one-way analysis of variances (ANOVA) with multiple comparisons between HC, SPMS, PPMS, or in case of only two groups, the two-tailed unpaired Student's *t-*test. To correct alpha-error inflation, a Bonferroni-based correction was applied.

## Results

### Ectopic Lymphoid Follicles in Progressive MS Are Rich in CXCR5^+^ Lymphocytes

To extend the knowledge about lymphocyte aggregates in the CNS of progressive MS patients (37), we evaluated extent, regional occurrence and especially germinal center (GC) characteristics on so far unexamined MS patients. Pre-defined chronic active lesion (CAL) and normal-appearing white matter (NAWM) tissue was screened blinded for infiltration based on H&E and scored by IF staining for lymphocytes (Table 1). 75% of CAL brain and 88% of CAL spinal cord cases, but no control cases, exhibited at least five infiltrated CD3^+^ and /or CD20^+^ lymphocytes. As the infiltration into NAWM/NASC (normal-appearing spinal cord) was negligible (only two regions, both score 1), we focused on tissue with chronic lesions for all following analyses. Median infiltration was determined by the mean of the previously defined scores per each case (Supplementary Figures 1G,H).

Putative regions with infiltrations were identified by H&E (Figures 1A,C), stained for CD3 and CD20 lymphocytes on serial sections (Figures 1B,D), and infiltration scores determined [adapted from Howell et al. (33)]. As reported before (31, 32), we found GC-like structures that are typically characterized by an accumulation of cells (Figures 1E,F), which could be identified as CD3^+^ T and CD20^+^ B cells (Figure 1G). We were also able to detect CD4^+^ cells, negative for CD138 (PCs; Figure 1H), but positive for CD3, specifying T-helper cells (Figure 1I). In addition, we found CD3^+^CD27^+^ memory T cells as well as CD3^+^CD8^+^ cells, the latter not co-staining for the memory marker CD27 (Figure 1P). Only few Ki67^+^ proliferating cells (Figure 1J), but many CD35^+^ (Figure 1K) as well as some CD21^+^ (Figure 1L) were present in these aggregates. CD35 and CD21 are commonly used to identify follicular dendritic cells (FDCs), but can also be expressed on B cells. CD68^+^ macrophages (Figure 1M) manifested ongoing inflammation. The presence of BCL-6^+^ (Figure 1N) and especially the abundance of CXCR5^+^ cells (Figure 1O) underlined the definition as eLFs.

### SPMS Is More Severely Affected Than PPMS and Exhibits eLFs-Defining Aggregates

**Figure 2:**
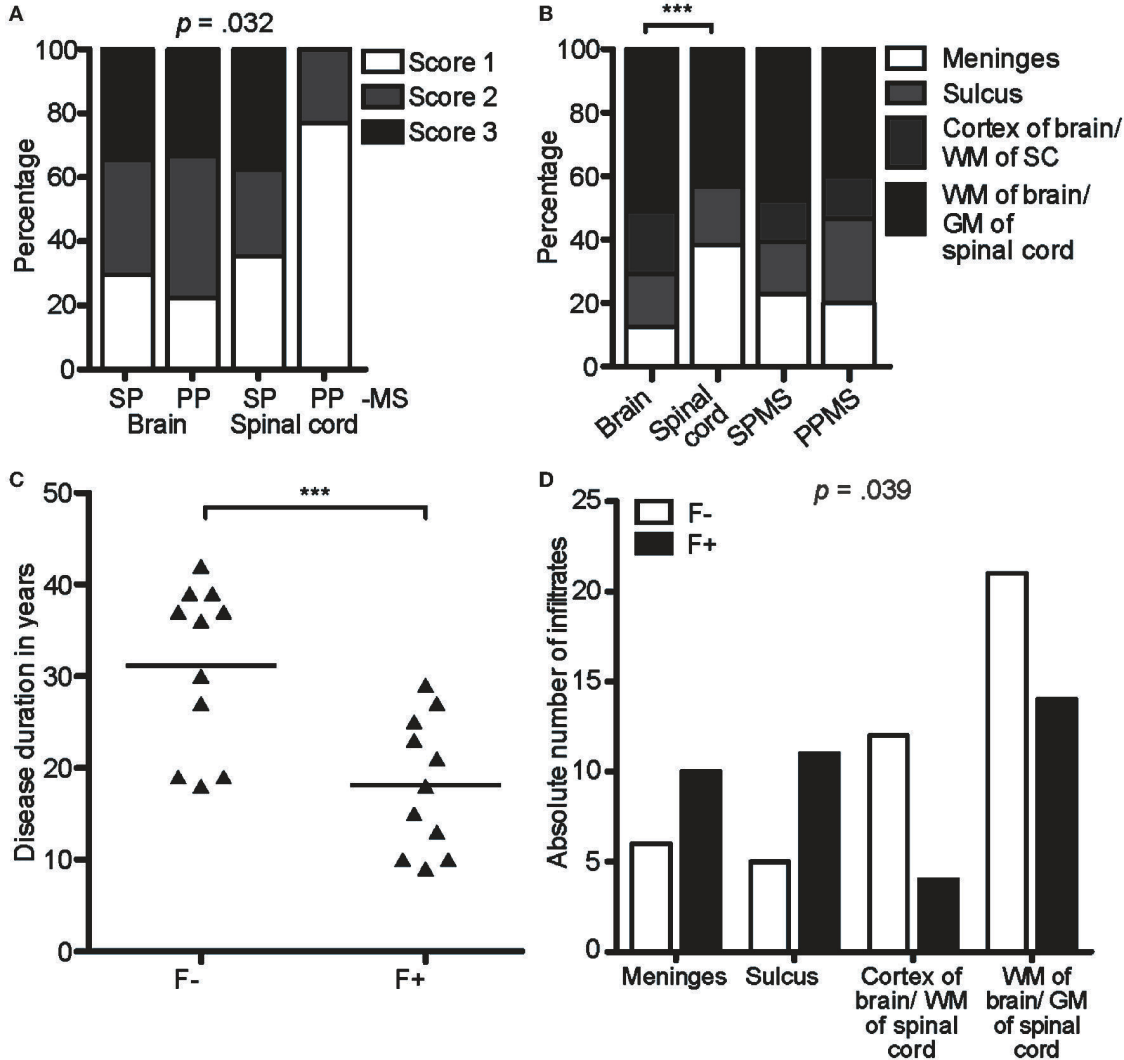
CXCR5-rich lymphoid aggregates are most prominent within meninges of SPMS patients. **(A)** Percentage of score infiltration in SPMS and PPMS in CAL brain and spinal cord. SPMS brain infiltrates, *n* = 78; SPMS spinal cord, *n* = 48; PPMS brain, *n* = 9; PPMS spinal cord, *n* = 13. Chi-square test, *X*^2^(6) = 13.77, *p* = 0.032, *d* = 0.641. **(B)** Percentage of regional infiltration in meninges, sulcus, cortex of the brain/WM of the spinal cord and parenchyma of the brain/GM of the spinal cord in CAL SPMS and PPMS. SPMS, *n* = 145; PPMS, *n* = 15; brain, *n* = 96; spinal cord, *n* = 64. Chi-square test for SPMS vs. PPMS, *X*^2^(6) = 1.02, *p* = 0.799, *d* = 0.168; Chi-square test for brain vs. spinal cord, *X*^2^(3) = 21.19, *p* < 0.001, *d* = 0.793. **(C)** Disease duration in years in follicle-like structures (F+) vs. other infiltrates (F-) in SPMS. F-, *M* = 31.18, *SD* = 9.05, *n* = 11; F+, *M* = 18.18, *SD* = 7.26, *n* = 11; unpaired *t*-test, *t*(20) = 3.72, *p* = 0.001. **(D)** Absolute number of infiltrates in meninges, sulcus, cortex of brain/WM of spinal cord, WM of brain/GM of spinal cord in follicle-like structures (F+) vs. other infiltrates (F-) of SPMS cases. F-, *n* = 44, F+, *n* = 39; *X*^2^(3) = 8.38, *p* = 0.039. ***p < 0.001.

To reveal any relevance for disease scores, we directly compared the infiltrates in primary and secondary progressive MS (PPMS and SPMS) patients (Figure 2A). Infiltrates of all scores were detected in patients with either form of progressive MS and scores of the brains of SPMS vs. PPMS exposed a similar score pattern. In the spinal cord sections, however, infiltrates tended toward more severe scores under SPMS (*X*^2^(6) = 13.77, *p* = 0.032, *d* = 0.641). Overall, we found a moderate effect in the distribution of regions between brain and spinal cord, tending toward more superficial infiltration (meninges and sulcus, brain: 29%; spinal cord: 56%) in spinal cord (*X*^2^(3) = 21.19, *p* < 0.001, *d* = 0.793), while the distribution pattern of the region of the infiltrates was similar between SPMS and PPMS (Figure 2B). Of note, we assume that in general the infiltration is more noticeable in the superficial regions as in the parenchyma, because the latter one encompasses a larger area than the meninges and sulcus.

To define lymphocyte aggregates as eLF /GC-similar, they had to reach score 3 and stain positively for CD3, CD20, Ki67, CD138, plus CD35/CD21 (39 found), thereafter termed F+. Besides FDCs also B cells express CD35 and /or CD21, but since CD35^+^ cells were usually more abundant than CD20^+^ B cells, we considered at least part of the CD35^+^ cells as FDCs. This interpretation is in line with former studies detecting such lymphoid aggregates (32). When the aggregates did not fulfill all criteria, but showed at least score 2, they were called F- (44 found). Interestingly, 50% (11/22) of the SPMS cases exhibited at least one GC-like structure (Table 1), whereas none could be detected in PPMS. As published before, but not reproduced in other studies, disease duration shortens from *M* = 31.18 years in F- cases to *M* = 18.18 years in F+ SPMS, pinpointing follicle-associated SPMS cases to more severe disease [*t*(20) = 3.72, *p* = 0.001, Figure 2C]. Age of death was similar between the patients of our cohort. The F+ eLFs in SPMS cases, but not the F-, were predominantly located within the meninges and sulci [*X*^2^(3) = 8.38, *p* = 0.039, d = 0.670, Figure 2D]. In sum, CXCR5-rich eLFs were most prominent within meninges and severity was enhanced in SPMS patients' spinal cord as compared to PPMS.

### No T_FR_s Exist in eLFs, Whereas NFATc1^+^ T_FH_s Are Enriched

**Figure 3:**
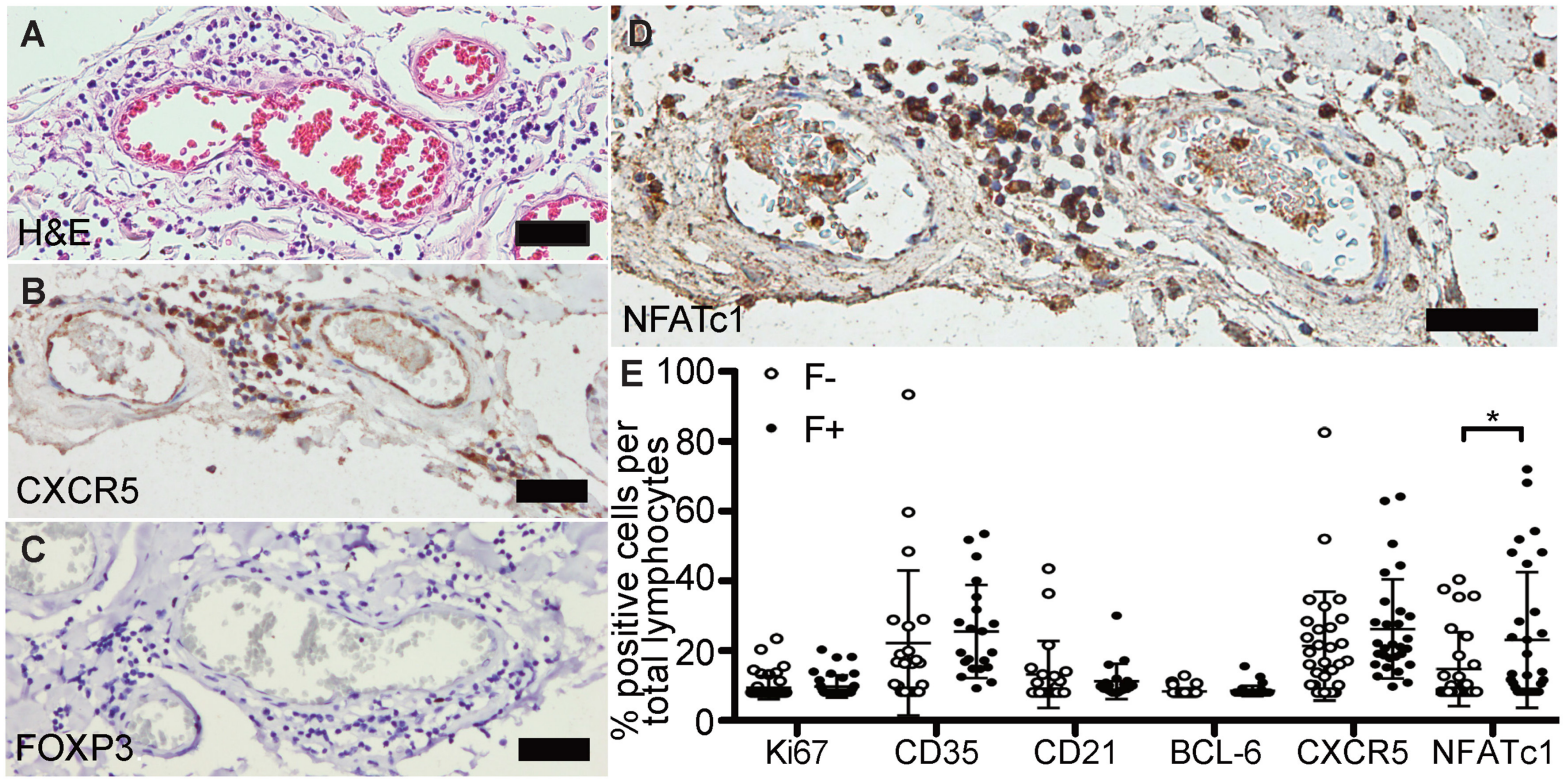
Follicle-like structures of SPMS brains are devoid of FOXP3 expression, but exhibit NFATc1^+^ cells. **(A)** Representative meningeal follicle-like structure of SPMS spinal cord, which was screened based on H&E staining and characterized by >60 lymphocytes (score 3), detection of Ki67^+^, CD35^+^/CD21^+^, and CD138^+^ cells on serial sections, termed F+. F–, if no or not all criteria were fulfilled. **(B)** Follicle-like structures could be characterized by CXCR5^+^, **(C)** but not by FOXP3^+^ cells. **(D)** NFATc1^+^ cells were present in follicle-like structures. **(E)** IHC stainings of Ki67, CD35, CD21, BCL-6, CXCR5, and NFATc1 were quantified as frequency of total cells in follicle-like structures (F+) and less defined infiltrates (F-). **Ki67**: F-, *M* = 1.52, *SD* = 3.73, *n* = 45; F+, *M* = 1.89, *SD* = 3.55, *n* = 38; Mann Whitney test, *U* = 734.0, *p* = 0.161. **CD35**: F-, *M* = 15.41, *SD* = 22.95, *n* = 22; F+, *M* = 19.05, *SD* = 14.75, *n* = 21; Mann Whitney test, *U* = 159.0, *p* = 0.081. **CD21**: F-, *M* = 5.68, *SD* = 10.63, *n* = 21; F+, *M* = 3.51, *SD* = 5.60, *n* = 20; Mann Whitney test, *U* = 183.5, *p* = 0.488. **BCL-6**: F-, *M* = 0.32, *SD* = 1.08, *n* = 44; F+, *M* = 0.49, *SD* = 1.62, *n* = 38; Mann Whitney test, *U* = 803.0, *p* = 0.642. **CXCR5**: F-, *M* = 14.72, *SD* = 17.24, *n* = 30; F+, *M* = 20.11, *SD* = 15.73, *n* = 29; Mann Whitney test, *U* = 319.0, *p* = 0.080. **NFATc1**: F-, *M* = 7.15, *SD* = 11.68, *n* = 28; F+, *M* = 16.36, *SD* = 21.46, *n* = 31; Mann Whitney test, *U* = 307.0, *p* = 0.043. Scale bars A-D indicate 100 μm. *, *p* < 0.05.

Next, we evaluated if the identified lymphoid aggregates in the CNS of SPMS patients could be properly controlled. Follicle-like structures were again screened on H&E (Figure 3A) and antibodies established on tonsils and follicular lymphoma of the spinal cord (Supplementary Figure 2). We found only 4/38 follicle-like structures positive for BCL-6, but each of them exhibited CXCR5 (Figure 3B). However, not a single FOXP3^+^ cell, i.e., a regulatory T cell was found (Figure 3C), although our control stainings were able to detect FOXP3^+^ cells (Supplementary Figures 2G,Q).

To reveal the phenotype of—unsuppressed—T_FH_ in eLFs, we focused on CXCR5 and NFATc1, which could be abundantly detected in follicle-like structures (Figure 3D). When comparing all markers between F- and F+ (Figure 3E), we detected an increase of percentage of CD35^+^ cells of total lymphocytes in F+ (*M* = 19.05) compared to F- infiltrates (*M* = 15.41). Rise in Ki67 and CD21 in F+ was only subtle compared to F- (Ki67, F-, *M* = 1.54; F+, *M* = 1.89; CD21, F-, *M* = 5.67; F+, *M* = 3.51). The amount of BCL-6^+^ cells per mm^2^ was very low in both, F- and F+, without any relevant differences. We found a small increase in CXCR5 in F+ (*M* = 14.72) compared to F- (*M* = 20.11), but with high inter-individual variations. Interestingly, we revealed a striking elevation in frequency of NFATc1^+^ cells per total lymphocytes in F+ (M = 16.36) compared to F- infiltrates (M = 7.15, *U* = 307.0, *p* = 0.043), pointing to more typical T_FH_ in F+ lymphoid aggregates.

### Detected CD4^+^ Cells Are CD3^+^ T Cells, but Neither Express PD-1 or Foxp3

**Figure 4:**
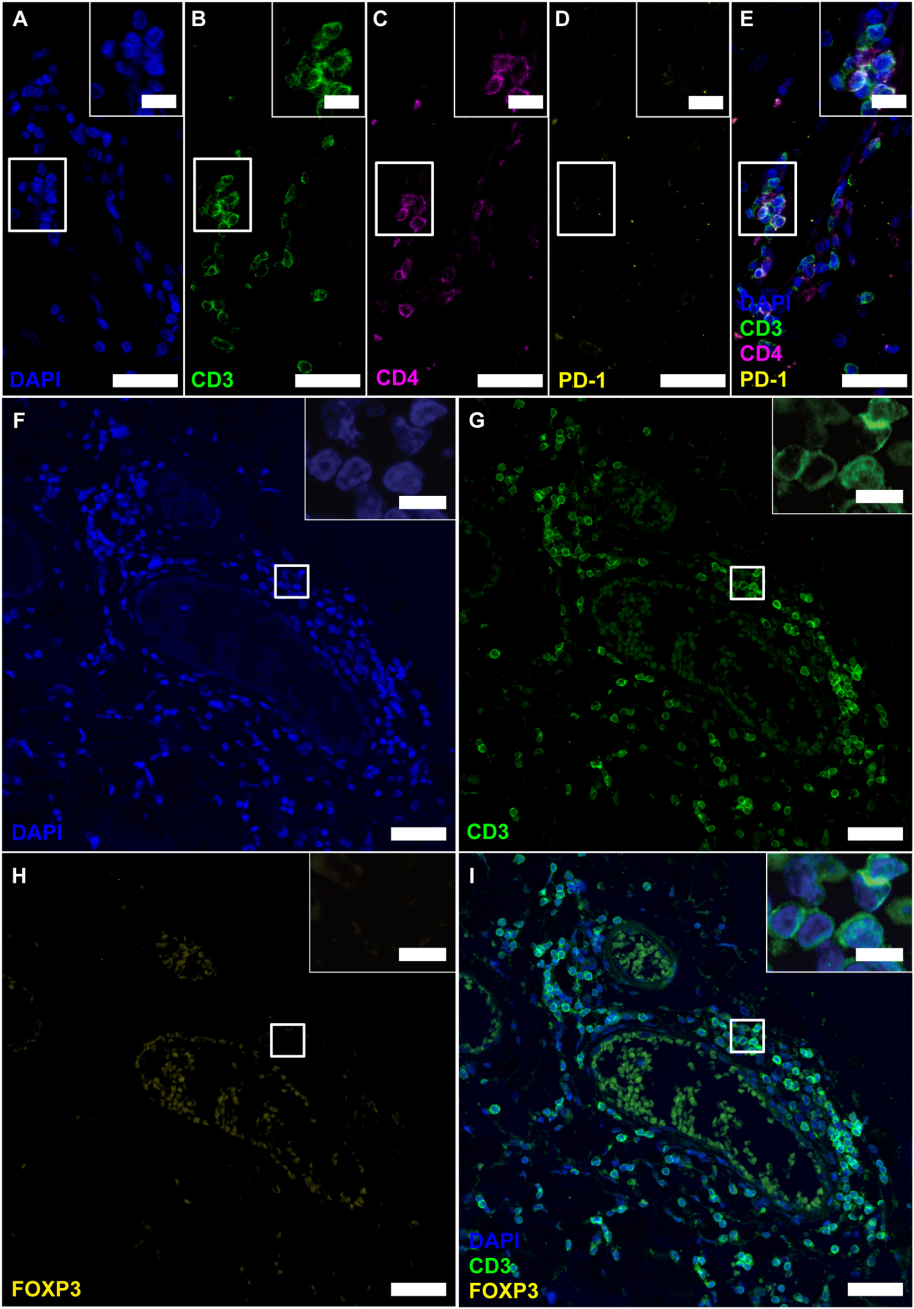
Follicle-like structures of SPMS brains exhibit CD3^+^CD4^+^ T cells, which neither express PD-1 nor FOXP3. **(A–E)** IF staining of CD3, CD4 and PD-1 reveal CD3^+^CD4^+^PD-1^−^ T-helper cells in progressive MS. Inserts in the upper right corners show magnification of the white box. **(F–I)** IF staining of CD3 and FOXP3 on serial sections of a representative meningeal follicle-like structure in SPMS (same region as Figure 3). CD3^+^ T cells, but no FOXP3^+^ cells were detected. Inserts show magnification of the white box. Scale bars indicate 100 μm, inserts 10 μm.

Before further investigating NFATc1 expression in follicular T cells, we wished to verify the CD4^+^ cells as T_FH_s /T_FR_s by simultaneous IF stainings of CD4 with CD3 and PD-1 (Figures 4A–E). Staining was established on tonsils, follicular lymphoma of the spinal cord and PCNSL of the brain (Supplementary Figures 3G–I). Although the presence of CD3 on all detected CD4^+^ cells clearly indicated T-cell identity, the complete absence of PD-1 questions the differentiation into T_FH_ as they emerge in GCs of secondary lymphoid organs. CD3^+^CD4^+^ T cells could also be Tregs. To again search for Tregs within the lymphoid aggregates, the same region as before (meningeal part of the spinal cord of SPMS patient) was screened on further serial sections for CD3 and FOXP3 co-expression (Figures 4F–I). Still, we could not detect any FOXP3^+^ cells, although Tregs were provable in human tonsils and follicular lymphoma (Supplementary Figures 3M,N), while not on PCNSL tissue (Supplementary Figure 3O).

### Foxp3^+^ Cells Are Sparse in the CNS of Progressive MS Patients

**Figure 5:**
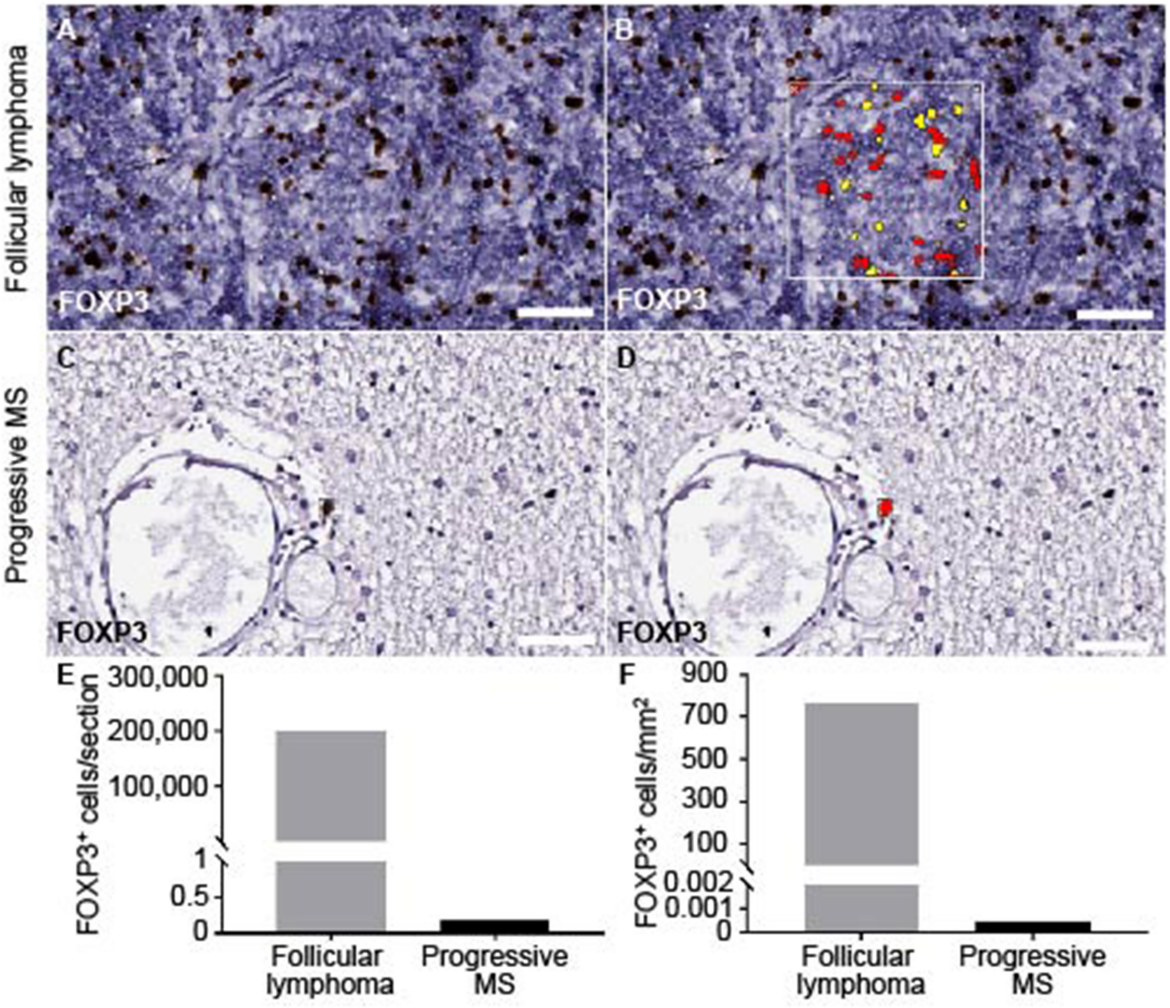
Quantification of FOXP3 staining on brain and spinal cord of progressive MS and follicular lymphoma. The module cytonuclear v1.6 (Halo, Indica Lab) was used to define FOXP3^+^ in follicular lymphoma **(A,B)** and applied on progressive MS tissue **(C,D)**. Representative raw images detecting FOXP3^+^ staining **(A,C)** and the mark-up (yellow, low positive; red, highly positive) are depicted **(B,D)**. **(E)** Absolute number of FOXP3^+^ cells per whole section in follicular lymphoma (*M* = 200849) and across progressive MS samples (*M* = 0.176, *n* = 36) as well as **(F)** FOXP3^+^ cells per tissue area in mm^2^ are counted in follicular lymphoma (*M* = 767.71) and across all available progressive MS samples (*M* = 0.00051, *n* = 36). Note, that in 83% of the samples (*n* = 30) no FOXP3^+^ cells were detected, in 11% samples (*n* = 4) one FOXP3^+^ cell within parenchyma, in 6% of samples 2 FOXP3^+^ cells within parenchyma. Scale bars indicate 50 μm.

The total absence of presumable T_FR_s was puzzling. Before and in line with literature, we had clearly detected CNS-resident Tregs in mice diseased with experimental autoimmune disease (39), and at least a few CD4^+^ FOXP3^+^ cells were found in early active brain lesions of human MS patients (40). To get an idea on the overall presence of Tregs in the CNS of progressive MS patients, we screened for FOXP3^+^ cells on the complete brain and spinal cord sections of our cohort. We chose an unbiased measure for cytoplasmic and nuclear staining and established the setting on Treg^hi^ follicular lymphoma located in the spinal cord (Figures 5A,B). Assuring, also the automated detection did not discover any FOXP3^+^ cells within the infiltrated areas. Furthermore, across the progressive MS tissue, we found at the most two FOXP3^+^ cells per section, always located in the parenchyma, but most patients' CNS emerged as entirely FOXP3 negative (Figures 5C,D). The machine-assisted counting revealed the striking difference per section or area between follicular lymphoma and progressive MS patients (Figures 5E,F). In sum, not only are Tregs absent in lymphoid aggregates, but also overall rare in the CNS of progressive MS patients.

### NFATc1 Is Predominantly Cytoplasmic in T_FH_ Cells

**Figure 6:**
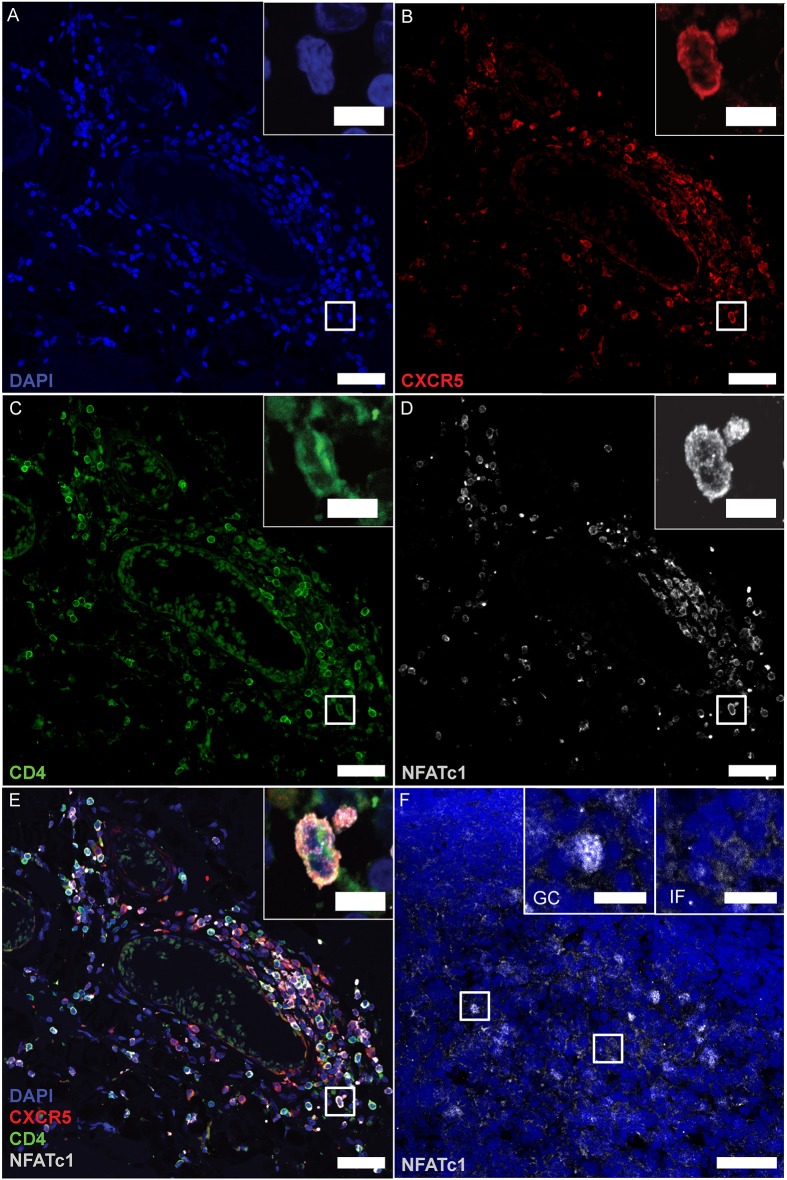
CD4^+^CXCR5^+^ T_FH_s mark positive for cytoplasmic NFATc1. **(A–E)** Consecutive IF staining of CD4, CXCR5 and NFATc1 on serial sections of follicle-like structures in SPMS (same region as Figures 3, 4E–H). Inserts show magnification of the white box. **(F)** NFATc1 appears to be cytoplasmic in MS brains, compared to nuclear localization within tonsillar GCs (left insert) and cytoplasmic predominance in inter-follicular cells (right insert). Scale bars indicate 100 μm, inserts 10 μm.

In follicular T cells of secondary lymphoid organs (SLOs), NFATc1 is highly expressed and nuclear, i.e., constitutively active, which in T_FR_s is necessary to upregulate CXCR5 and home to GCs (17). To tackle the follicular nature of CD4^+^ T cells in the lymphoid aggregates, we performed IF stainings of CD4, CXCR5 together with NFATc1, again after having established detection on tonsils, follicular lymphoma of the spinal cord, and PCNSL of the brain (Supplementary Figures 3P–R). We found CD4- and CXCR5-single positive cells and a good number of CD4^+^CXCR5^+^ cells. Here, clear NFATc1 expression resembled CD4^+^CXCR5^+^ T_FH_s (Figures 6A–E). Unexpectedly, however, NFATc1 was located within the cytoplasm, indicating a less active state. In SLOs like tonsils, cytoplasmic NFATc1 characterized inter-follicular cells, whereas NFATc1 appeared nuclear, i.e., activated, within GCs (Figure 6, upper right inlays). Taken together, lymphoid aggregates were devoid of T_FR_s as well as PD-1^+^ T_FH_s, while the augmented expression of NFATc1 in CD4^+^CXCR5^+^ T_FH_-like cells was restricted to the cytoplasm.

### The Predominance of B Cells Is a Hallmark of eLFs

**Figure 7:**
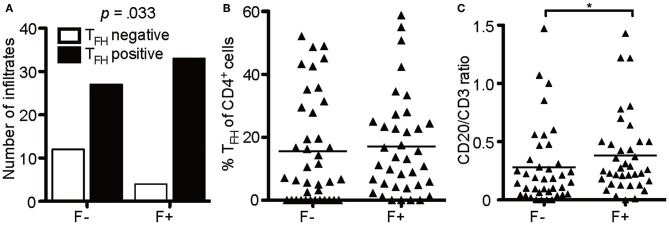
B cells enrich in lymphoid aggregates. **(A)** Absolute number of infiltrates that were positive for T_FH_ in follicle-like structures (F+) and less defined infiltrates (F-). Fisher's exact test, *N* = 76, *X*^2^(1) = 4.55, *p* = 0.048, *d* = 0.505. **(B)** Mean percentage of T_FH_ cells defined as CD4^+^CXCR5^+^ cells of CD4^+^ cells in two serial FFPE sections of follicle-like structures (F+) and less defined infiltrates (F-) in SPMS brains and spinal cords. F-, *M* = 15.57, *SD* = 17.13, *n* = 39; F+, *M* = 17.04, *SD* = 15.85, *n* = 37. Mann Whitney test, *U* = 635.0, *p* = 0.369. **(C)** CD20/CD3 ratio in follicle-like structures (F+) and less defined infiltrates (F-) based on IF co-staining of CD3 and CD20. F-, *M* = 0.28, *SD* = 0.33, *n* = 39; F+, *M* = 0.38, *SD* = 0.34, *n* = 37; Mann Whitney test, *U* = 525.5, *p* = 0.042. *p < 0.05.

Of note, not every follicle-like structure exhibited CD4^+^CXCR5^+^ T cells (only 78%), but the number of infiltrates was moderately associated with follicle-like structures [Figure 7A, *X*^2^(1) = 4.55, *p* = 0.048, *d* = 0.505]. However, the mean percentage of CD4^+^CXCR5^+^ T cells of total CD4^+^ cells in two stainings did not show any relevant difference between F- (*M* = 15.57) and F+ (*M* = 17.01) (Figure 7B). To propose the source of heightened cell numbers in F+, we found a moderate increase in CD20/CD3 ratio in F+, indicating the supremacy of CD20^+^ B cells in those lymphoid aggregates (Figure 7C, F-, *M* = 0.279; F+, *M* = 0.381, *U* = 525.5, *p* = 0.042). Therefore, the presumable eLFs exhibited not only an increased number of CD35^+^ cells, likely FDCs, and T_FH_-like cells with cytoplasmic NFATc1, but also a boosted CD20/CD3 ratio.

### CD4^+^CD69^+^ Cells Are Enriched in eLFs

**Figure 8:**
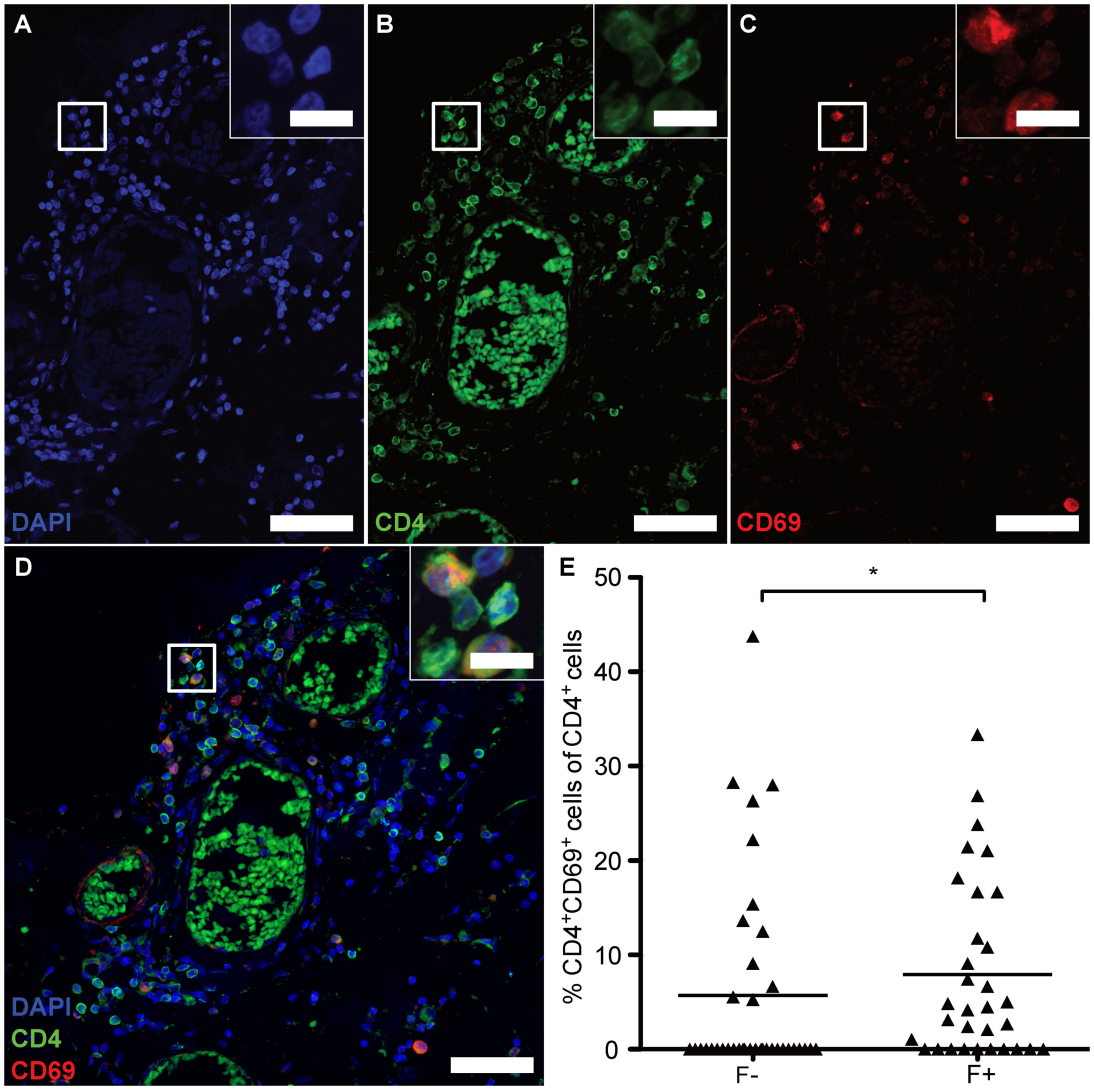
eLFs of brain and spinal cord exhibit more CD4^+^CD69^+^ cells. **(A–D)** Consecutive IF co-staining of CD4 and CD69 in follicle-like structures of SPMS brains and spinal cords. Inserts show co-localization of CD4^+^ cells with CD69 suggesting tissue-resident T cells in a representative meningeal eLF of SPMS spinal cord (same region as Figures 3, 4E–H, 5). Scale bar indicate 100 μm, scale bars of the inserts indicate 10 μm. **(E)** Percentage of tissue-resident cells defined as CD4^+^CD69^+^ cells of CD4^+^ cells in follicle-like structures (F+) and less defined infiltrates (F-) in SPMS brains and spinal cords. F-, 5.70, *SD* = 10.67, *n* = 38; F+, *M* = 7.92, *SD* = 9.39, *n* = 32; Mann Whitney test, *U* = 434.0, *p* = 0.028.

Absence of Tregs /T_FR_s and heightened B-cell frequency would predict an uncontrolled, overactive GC reaction. Nevertheless, CD4^+^CXCR5^+^ T cells exhibited NFATc1 in the cytoplasm and expressed only little BCL-6 and no PD-1, wherefore the lymphoid aggregates might meanwhile be the source for memory cells (41). Hence, we lastly studied whether follicles were associated with CD4^+^CD69^+^ cells, suggesting tissue-resident memory CD4^+^ T cells (T_RM_). Since we verified the identity of CD4^+^ to be CD3^+^ T cells on one example of eLF (Figures 4A–D) and the presence of CD3^+^CD27^+^, but CD8^−^ memory T cells (Figure 1P), putative T_RM_ helper cells were identified by co-staining of CD4 and CD69 on serial sections (Figures 8A–D). Indeed, we found a moderate increase in percentage of CD4^+^CD69^+^ cells in the total amount of CD4^+^ cells in F+ (*M* = 7.92) when compared to F- (*M* = 5.70) (Figure 8E, *U* = 434.0, *p* = 0.028). This finding indicates that the lymphoid aggregates /eLFs in the CNS of SPMS patients eventually became a source not only for LLPCs most likely producing auto-antibodies, but also most likely for memory T_FH_ and CD4^+^ T_RM_s that might account for the less active state of T_FH_ in spite of the absence of T_FR_ cells.

## Discussion

Being intrigued by the detection of lymphoid aggregates, which resemble germinal centers (GCs), in post-mortem material of progressive MS patients (31, 32) and knowing about the relevance of follicular regulatory T-cells (T_FR_s) in GCs (17, 42), we set out to inquire if those ectopic lymphoid follicles (eLFs) inhabit T_FR_s. In fact, also in this new cohort of progressive MS patients, we could find eLFs—defining them by the combined presence of CD3^+^ T cells, CD20^+^ B cells, Ki67^+^ proliferating, CD35^+^ or CD21^+^ (FDCs?) cells and parafollicular CD138^+^ PCs as well as a minimum of 61 lymphocytes—in around half of all SPMS cases. However, no Tregs and therefore no T_FR_s could be detected in eLFs of SPMS patients or in lesser-defined lymphoid aggregates of PPMS and SPMS patients. In GCs of secondary lymphoid organs (SLOs), T_FR_s repress T_FH_ and GC-B cells (15, 18–22). Accordingly, the total absence of T_FR_s and the succeeding unrestrained T-cell help to low-affinity, poly-reactive or even autoreactive B cells must lead to overshooting GC reactions and the occurrence of CNS-specific autoantibodies in MS patients. In line, PCs have been detected scattered in the CNS parenchyma of remitting-relapsing and secondary progressive MS patients, but in SPMS patients predominantly at the periphery of B-cell follicles /eLFs (31, 43).

It is increasingly clear that loss of Treg number and /or function is implicated in a wide variety of autoimmune and chronic inflammation settings. If this is due to loss of FOXP3 expression, those cells are called ex-Tregs and they have been linked to susceptibility for MS (44). Since preliminary experiments (data not shown) indicated some circulating CD4^+^CXCR5^+^FOXP3^+^ cells in lumbar punctures, i.e., the CSF of living SPMS patients, it is possible that such T_FR_ cells lose FOXP3 expression—becoming ex-T_FR_s—and their suppressive capacity when homing to eLFs. In general, the scarcity of Tregs in the CNS of progressive MS patients (our data here) as well as in early active MS lesions is in contrast with the detectability of some Tregs in the CSF (40). The pro-inflammatory cytokine milieu might be causative, as for example high levels of IL-6 can induce loss of FOXP3 expression of Tregs *in vivo* (45, 46). Interestingly, dysfunctional ex-T_FR_ cells have been described in an animal model just now (47), but nothing is known about human ex-T_FR_s, what would lead to FOXP3 downregulation and what could be the consequence.

Experimental autoimmune encephalomyelitis (EAE), a rodent model sharing features with MS, revealed not only that the pro-inflammatory CCR6-directed Th17 cells enter the CNS via the choroid plexus, a distinct meningeal structure (48), but also are involved in eLF induction (49, 50). Strikingly, both Th17 and Foxp3^+^ T cells can acquire T_FH_ cell-like characteristics when migrating to mouse Peyer's Patches of the intestine (51, 52). Whether the inflamed CNS in humans provides environmental cues for transdifferentiation of Th17 as well as Foxp3^+^ T cells to T_FH_s stays unresolved. At least, both CD4^+^ T-cell types display a great magnitude of plasticity with pathogenic potential in humans (53).

Before, we had shown that encephalitogenic T cells rely on NFATc1 and NFATc2 expression and activity (39). Furthermore, the high abundance of NFATc1 expression in lymphoid aggregate-situated T_FH_s was reminiscent of GCs in mice and men (16, 17), describing eLFs now better than less defined lymphoid aggregates. In contrast to constitutive nuclear expression of NFATc1 in GCs (17), however, NFATc1 was mostly cytoplasmic and presumably inactive like in interfollicular T cells of SLOs. Since NFATc1 transactivation is necessary for the induction of CXCR5 in T_FR_s (17), they could lose CXCR5 expression in such a scenario and not—or no longer—be present in eLFs.

Nevertheless, cytoplasmic NFATc1 is untypical for classical T_FH_s and better describes interfollicular T cells (17). Chronic stimulation of CD8^+^ or CD4^+^ T cells leads to exhaustion and anergy, respectively, a measure to protect the organism from unlimited immune responses. In both T-cell types, NFAT proteins, being directly downstream of T-cell receptor (TCR) signaling, are key to the response (54, 55). We showed in a mouse model of chronic LCMV infection that restraining the TCR → NFATc1 → IRF4 axis by IRF4 heterozygosity redirected exhausted CD8^+^ T cells to memory-like CXCR5^+^CD8^+^ T cells (56). In the CNS of SPMS patients, we found CD3^+^CD8^−^CD27^+^ memory T cells and especially CD3^+^CD4^+^CXCR5^+^BCL6^lo/−^ PD-1^−^ T_FH_ cells. The latter is the phenotype of circulating memory T_FH_ cells (16, 41, 57). Intriguingly, also primary T_FH_s of SLOs persist as memory T cells in the outer follicle after the collapse of the GC (58). Upon re-exposure with the antigen, they expand within reactive follicles and spread via the lymphatic flow. In the CNS, such CXCR5^+^ memory T_FH_ and T_RM_ cells could disseminate via lymphatic vessels, which connect the cerebrospinal fluid to the deep cervical lymph nodes (59). This setting enables a smoldering humoral immune response over the entire CNS.

The presence of—uncontrolled—lymphoid aggregates in proximity to the meninges is in agreement with the assumption that those structures promote auto-antigen-specific adaptive immune responses that exacerbate chronic disease. Again, different from GCs of SLOs and more like a primary follicle or a collapsed eLF with memory T_FH_ cells, we found very limited expression of the transcriptional key regulator BCL-6 as well as only few Ki67^+^ proliferating cells, suggesting that they are in a resting state. In addition, CD21^+^ cells were sparse and the CD35^+^ did not abundantly reach-out to a typical FDC network. Some of them might even be GC-B cells or FDC precursors (60, 61). Actually, the role of FDCs can be fulfilled by other cell types in eLFs, like either monocytes/macrophages or fibroblasts produce CXCL13 under eLF forming conditions (62, 63). This all is in line with the fact that fully developed eLFs with FDCs, compartmentation into a light and dark zone, an excess of GC-B cells over T_FH_s as well as the presence of “true”, i.e., active CD4^+^CXCR5^+^BCL-6^+^ T_FH_s are rare in human autoimmune diseases (64). Since T cells of inflamed tissues can still provide cognate help to GC–B cells in unstructured, FDC-negative infiltrates, we envisage a transient conversion from F- to F+ eLFs, the latter composed with FDCs and CXCR5^+^ T_FH_s in SPMS. Of note, the relative frequency of CD4^+^CXCR5^+^ T_FH_s was similar in less defined (F-) aggregates as in eLFs, suggesting that GC-like reactivity is hallmark in the CNS of progressive forms of MS. Still, at time of death, germinal center reactivity might have come to a halt. Nonetheless, this leads to the assumption that for both progressive forms, SPMS and PPMS, therapeutic targeting of B or T_FH_ cells could be promising. An awareness for intrathecal LLPCs (4) and T_RM_s increases the treatment options. At last, finding a way to revive Tregs /T_FR_s in the inflamed CNS promises so far unappreciated benefits.

## Data Availability Statement

All datasets generated for this study are included in the article/Supplementary Material.

## Ethics Statement

The studies involving human participants were reviewed and approved by the Multicentre Research Ethics Committee (08/MRE09/31). The patients/participants provided their written informed consent to participate in this study.

## Author Contributions

LB designed the study, performed research, analyzed and discussed the data, and took major part in writing the manuscript. AL performed research. AR discussed the data and provided financial support. CM supported the experiments, discussed the design as well as the data of the study. FB-S conceptualized the research goals, acquired major funding, designed research, discussed the data, and wrote the manuscript.

### Conflict of Interest

The authors declare that the research was conducted in the absence of any commercial or financial relationships that could be construed as a potential conflict of interest.
