# Supplementary material for: Lymphoid Aggregates in the CNS of Progressive Multiple Sclerosis Patients Lack Regulatory T Cells
Source: Front Immunol. 2020 Jan 15;10:3090. doi: 10.3389/fimmu.2019.03090 (PMC6974514; doi:10.3389/fimmu.2019.03090)
Supplement: Supplementary file 1 [file Data_Sheet_1.PDF]

Supplementary Material

Lymphoid aggregates in the CNS of progressive multiple sclerosis patients lack regulatory T cells

Luisa Bell[1], Alexander Lenhart[1], Andreas Rosenwald[1,2], Camelia M. Monoranu[3], Friederike Berberich-Siebelt[1*]

[1]Institute of Pathology, Julius-Maximilians University of Wuerzburg, Wuerzburg, Germany

[2]Comprehensive Cancer Center Mainfranken, Julius-Maximilians University of Wuerzburg, Wuerzburg, Germany

[3]Institute of Pathology, Department of Neuropathology, Julius-Maximilians University of Wuerzburg, Wuerzburg, Germany

*** Correspondence:**
Friederike Berberich-Siebelt
path230@mail.uni-wuerzburg.de

Supplementary Table 1. Demographic and clinical data of single cases

Case	Disease status / Diagnosis	Sex	Age at death	Cause of death	Age at disease onset	Disease duration, years	Death to TP, hours	No of relapses	Treatment	
									Relapses	DMT
MS 136	SPMS	m	40	Respiratory failure, sepsis, multiple sclerosis	31	9	10	10	ivMP	+
MS 166	SPMS	f	52	Bronchopneumonia, multiple sclerosis	16	36	7	8	ACTH	+
MS 169	SPMS	f	80	Septicemia	38	42	10	10	ACTH	n/a

MS 179	SPMS	f	70	Aspiration pneumonia, sepsis, multiple sclerosis	n/a	n/a	20	2	Carbama-zepine	+
MS 180	SPMS	f	44	Multiple sclerosis	34	>10	9	10	ivMP	n/a
MS 186	SPMS	f	58	Multiple sclerosis	21	37	24	10	ACTH	+
MS 200	SPMS	f	44	Urinary tract infection, sepsis, multiple sclerosis	25	19	20	5	ivMP	n/a
MS 201	PPMS	m	43	Strangulation, suicide	28	15	28	1	n/a	n/a
MS 202	SPMS	f	58	Pulmonary embolism, deep vein thrombosis	35	23	39	5	n/a	n/a
MS 204	SPMS	m	58	Chronic monomyelocytic leukemia, multiple sclerosis	39	19	35	11	ivMP, ACTh, steroids	n/a
MS 207	SPMS	f	46	Pneumonia, multiple sclerosis	21	25	10	4	ivMP	n/a
MS 211	SPMS	f	48	Pneumonia, multiple sclerosis	33	15	47	2	n/a	n/a
MS 293	SPMS	f	53	Multiple sclerosis	35	18	44	10	ivMP	+
MS 313	PPMS	m	66	Gastrointestinal bleeding caused by peptic ulcer disease, multiple sclerosis	37	29	16	1	n/a	ACTH
MS 325	PPMS	m	51	Bronchopneumonia	49	2	13	1	n/a	n/a
MS 330	SPMS	f	59	Pneumonia, multiple sclerosis	20	39	21	20	ACTH, ivMP	n/a
MS 338	SPMS	f	50	Pulmonary hemorrhage due to pulmonary embolism, severe multiple sclerosis	37	13	26	1	n/a	n/a
MS 340	SPMS	f	53	Sepsis, aspiration pneumonia, multiple sclerosis, perinephric abscess	34	19	17	5	n/a	n/a
MS 363	PPMS	m	42	End stage of Multiple sclerosis, respiratory failure	15	27	20	1	n/a	+
MS 383	PPMS	m	42	Aspiration pneumonia, multiple sclerosis	34	8	17	1	ivMP	n/a
MS 386	PPMS	f	90	Pneumonia, pulmonary fusion, pulmonary aneurysm	31	59	9	3	n/a	n/a
MS 389	SPMS	f	55	End stage of multiple sclerosis, multiple urinary sepsis	28	27	14	8	Dexa-methasone, ivMP	
MS 408	SPMS	m	39	Pneumonia, sepsis	29	10	21	8	ivMP	+
MS 473	PPMS	f	39	Bronchopneumonia, multiple sclerosis	26	13	9	1	ivMP	n/a
MS 478	SPMS	f	63	Metastatic bowel cancer, multiple sclerosis	24	39	24	6	ivMP	n/a
MS 485	PPMS	f	57	Bronchopneumonia, advanced multiple sclerosis	28	29	24	1	n/a	n/a

MS 489	SPMS	f	76	Cerebrovascular disease, multiple sclerosis	39	37	9	2	n/a	n/a
MS 492	PPMS	f	66	Sigmoid cancer	35	31	15	n/a	n/a	n/a
MS 494	PPMS	f	65	End stage of multiple sclerosis, chest infection	31	34	25	2	n/a	n/a
MS 497	SPMS	f	60	Aspiration pneumonia	31	29	10	5	Carbama-zepine	n/a
MS 500	PPMS	m	50	Urinary sepsis	21	29	7	n/a	n/a	n/a
MS 503	SPMS	f	53	Bronchopneumonia, osteomyelitis, acute pancreatitis, gallstone obstruction, multiple sclerosis	23	30	10	2	n/a	n/a
MS 504	SPMS	f	50	Aspiration pneumonia	23	27	20	4	ivMP	n/a
MS 513	SPMS	m	51	Multiple sclerosis, respiratory failure	33	18	17	4	n/a	n/a
C 022	Mild ageing-related changes	f	69	Lung cancer	-	-	33	-	-	-
C 025	no pathological changes in brain	m	35	Carcinoma of tongue	-	-	22	-	-	-
C 032	age-related changes, no malignancy	m	88	Prostate cancer, bone metastases	-	-	22	-	-	-
C 036	age-related changes, no malignancy	m	68	Cor pulmonale heart failure, fibrosing alveolitis, coronary artery atheroma			30	-	-	-
C 037	age-related changes, no malignancy	m	84	Bladder cancer, pneumonia			5	-	-	-
--C 0--39	age-related changes, no malignancy	m	82	Myelodysplastic syndrome, rheumatoid arthritis			21	-	-	-
-C 045	Mild ageing-related changes	m	77	Cardio pulmonary degeneration, prostate cancer, old age, Alzheimer's disease			22	-	-	-
C 048	micro-vascular pathology	m	68	Metastatic colon cancer			10	-	-	-
C 052	Mild ageing-related changes	m	70	Metastatic mixed sarcoma, pulmonary emboli and myocardial infarction			47	-	-	-
C 054	Mild ageing-related changes and mild hypoxia-	m	66	Pancreatic cancer			16	-	-	-

	related features									
C 059	age-related changes, no malignancy	m	86	Myocardial infarction			38	-	-	-
C 064	multiple cerebellar infarcts and microvascular pathology of the basal ganglia	m	63	Multiple strokes, acute renal failure, pneumonia			21	-	-	-
PD 032	ageing-related changes and microvascular pathology: small infarct in the putamen	f	91	not reported			n/a	-	-	-
PD 034	mild age-related and microvascular changes	m	90	Respiratory failure secondary to bronchial cancer			n/a	-	-	-

f, female; m, male
NAWM, normal appearing white matter
CL, chronic lesion
NASC, normal appearing spinal cord
CLSC, chronic lesional spinal cord
PD, Parkinson's disease
ACTH, adrenocorticotropin hormone
ivMP, intravenous methylprednisolone
Death to TP, death-to-tissue preservation interval
DMT, disease-modifying treatment
n/a, not applicable

Supplementary Table 2. Demographic and clinical data of groups

	Control cases	PPMS	SPMS	Test statistics
Sex	1 female = 8%<br>11 male = 92 %<br>F/M = 0.09	5 female = 45%<br>6 male = 55%<br>F/M = 0.83	19 female = 86%<br>3 male = 14 %<br>F/M = 6.3	$X^2(1)=6.188$,<br>*p* = 0.013, d=0.96
Death-to-tissue interval	*Mdn* = 22<br>*IQR* = 15	*Mdn* = 17<br>*IQR* = 12	*Mdn* = 20.5<br>*IQR* = 18	*ns*
Age of death	HC:<br>*Mdn* = 69.5<br>*IQR* = 17<br>PD:<br>*Mdn* = 90.5<br>*IQR* = 1	*Mdn* = 51<br>*IQR* = 24	*Mdn* = 53<br>*IQR* = 11.75	$F(2, 44) = 10.47$,<br>$p < 0.001$<br>HC *vs.* PPMS:<br>$t(23) = 3.50$, $p = 0.006$<br>HC *vs*. SPMS:<br>$t(34) = 4.33$, $p < 0.001$
Age of disease onset		*Mdn* = 31<br>*IQR* = 9	*Mdn* = 31<br>*IQR* = 12	*ns*
Disease duration		*Mdn* = 29<br>*IQR* = 18	*Mdn* = 24<br>*IQR* = 19	*ns*
Brain weight*	Not reported	*M* = 1270<br>*SD* = 179.2	*M* = 1133<br>*SD* = 109.7	$t(30) = 2.677$, $p = 0.012$
CSF pH**	Not reported	*Mdn* = 6.85<br>*IQR* = 1.02	*Mdn* = 6.95<br>*IQR* = 0.68	*ns*

* PPMS, *n* = 11/11; SPMS *n* = 21/22;
** PPMS, *n* = 4/11; SPMS *n* = 8/22

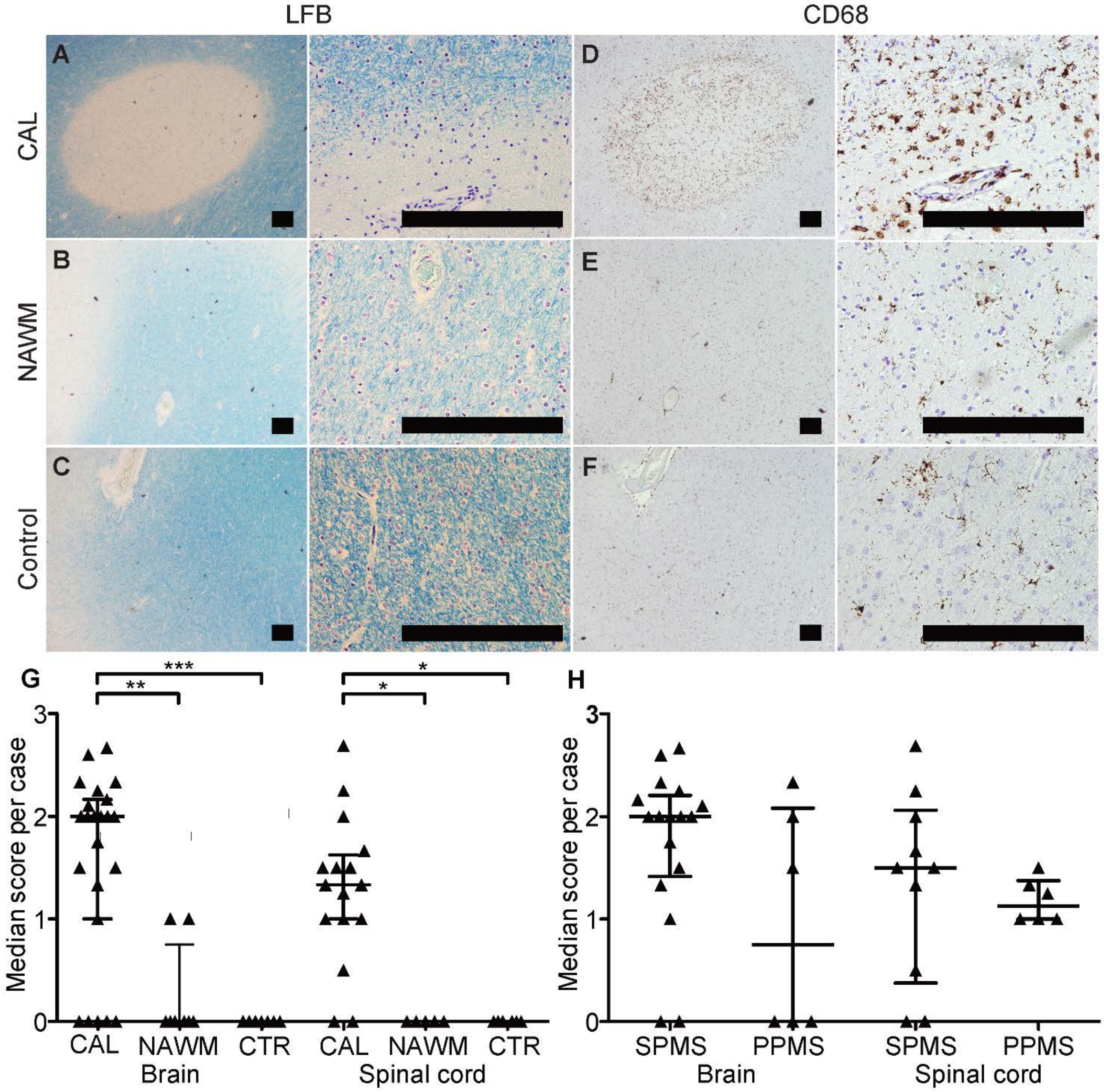

Supplementary Figure 1. Tissue characterization in CAL and NAWM progressive MS and control cases. **(A)** LFB staining shows a clear area of demyelination in CAL brain, whereas myelin sheath is intact in NAWM **(B)** and control brains **(C)**. (**D)** IHC shows a massive accumulation of $CD68^{+}$ macrophages in CAL brain, but minor infiltration of $CD68^{+}$ cells in NAWM (**E**) and negligible amount of macrophages in control brain **(F)**. **(G)** Infiltrated regions were screened on H&E staining and consecutively stained for $CD3^{+}$ and $CD20^{+}$ lymphocytes. Every infiltrated region was scored as followed: score 1, <30 lymphocytes; score 2, >31<60 lymphocytes; score 3, >60 lymphocytes. Median score per case was then compared between tissue types by Kruskal-Wallis test followed by Dunn-Bonferroni-Tests: $F(5, 59) = 33.74$, $p < .001$; MS CAL brain, $Mdn = 2.0$, $IQR = 1.66$, $n = 23$; MS NAWM brain, $Mdn = 0$, $IQR = 0.75$, $n = 8$; control brain, $Mdn = 0$, $IQR = 0$, $n = 7$; MS CAL spinal cord, $Mdn = 1.33$, $IQR = 1.69$, $n = 16$; NAWM spinal cord, $Mdn = 0$, $IQR = 0$, $n = 5$; control spinal

cord, *Mdn* = 0, *IQR* = 0, *n* = 6. MS CAL brain *vs* MS NAWM brain, $p = .002$; MS CAL brain *vs* control brain, $p = .001$; MS NAWM brain *vs* control brain, $p = .202$; MS CAL spinal cord *vs* MS NAWM spinal cord, $p = .004$; MS CAL spinal cord *vs* control spinal cord, $p = .002$; MS NAWM spinal cord *vs* control spinal cord, $p = .999$. **(H)** Median score per case was compared between SPMS *vs* PPMS in brain and spinal cord by Kruskal-Wallis test: $F(3, 35) = 5.99$, $p = .112$; CAL SPMS brain, *Mdn* = 2, *IQR* = 0.79, *n* = 17; PPMS CAL brains, *Mdn* = 0.75, *IQR* = 2.1, *n* = 10; SPMS CAL spinal cord; *Mdn* = 1.5, *IQR* = 1.7, *n* = 10; PPMS CAL spinal cord, *Mdn* = 1.13, *IQR* = 0.38, *n* = 6. SPMS brain *vs* PPMS brain, $p = .155$, SPMS spinal cord *vs* PPMS spinal cord, $p = .325$. Scale bars indicate 500 µm, LFB, Luxol fast blue; NAWM, normal-appearing white matter; CAL, chronic active lesion; *, $p < 0.05$; **, $p < 0.01$; ***, $p < 0.001$.

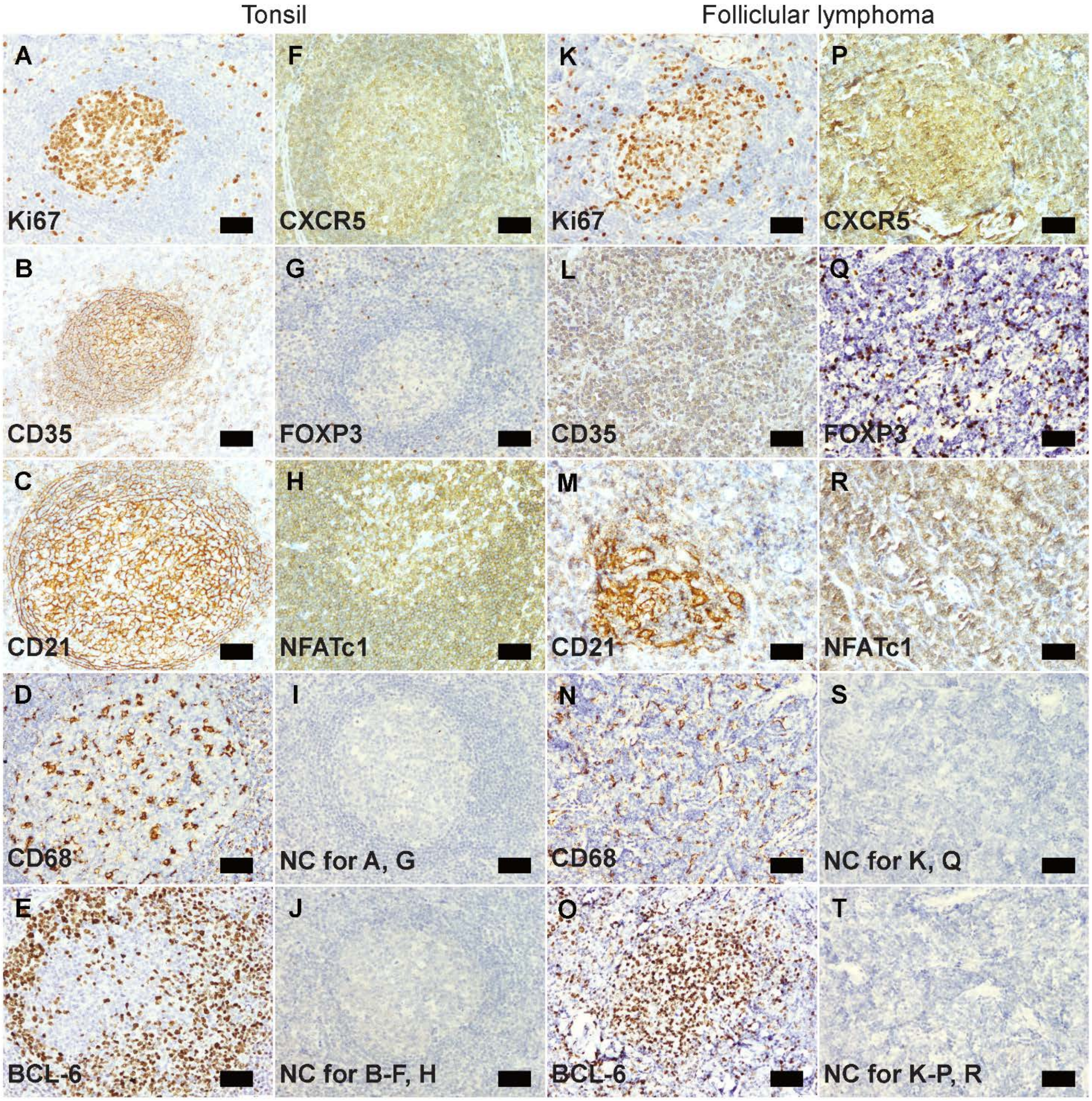

Supplementary Figure 2. IHC control stainings of tonsil and follicular lymphoma. FFPE sections of tonsils **(A-H)** and follicular lymphoma **(K-R)** were stained for Ki67, CD35, CD21, CD68, BCL6, CXCR5, FOXP3 and NFATc1. Negative controls were stained with secondary antibody only in tonsils **(I, J)** and follicular lymphoma **(S, T)**. NC, negative control. Scale bars indicate 100 µm.

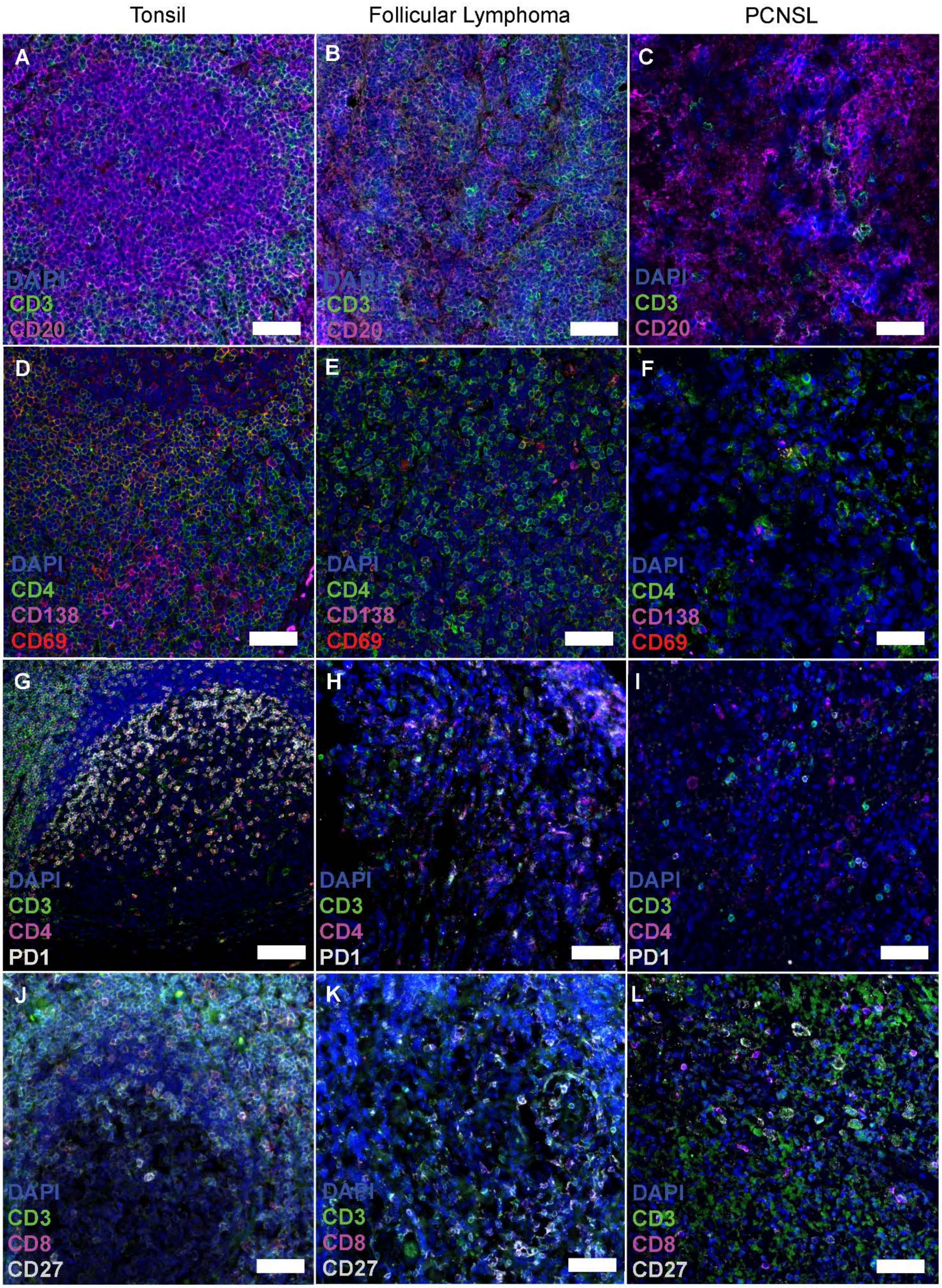
Tonsil
Follicular Lymphoma
PCNSL
A
B
C
DAPI
CD3
CD20
D
E
F
DAPI
CD4
CD138
CD69
G
H
I
DAPI
CD3
CD4
PD1
J
K
L
DAPI
CD3
CD8
CD27

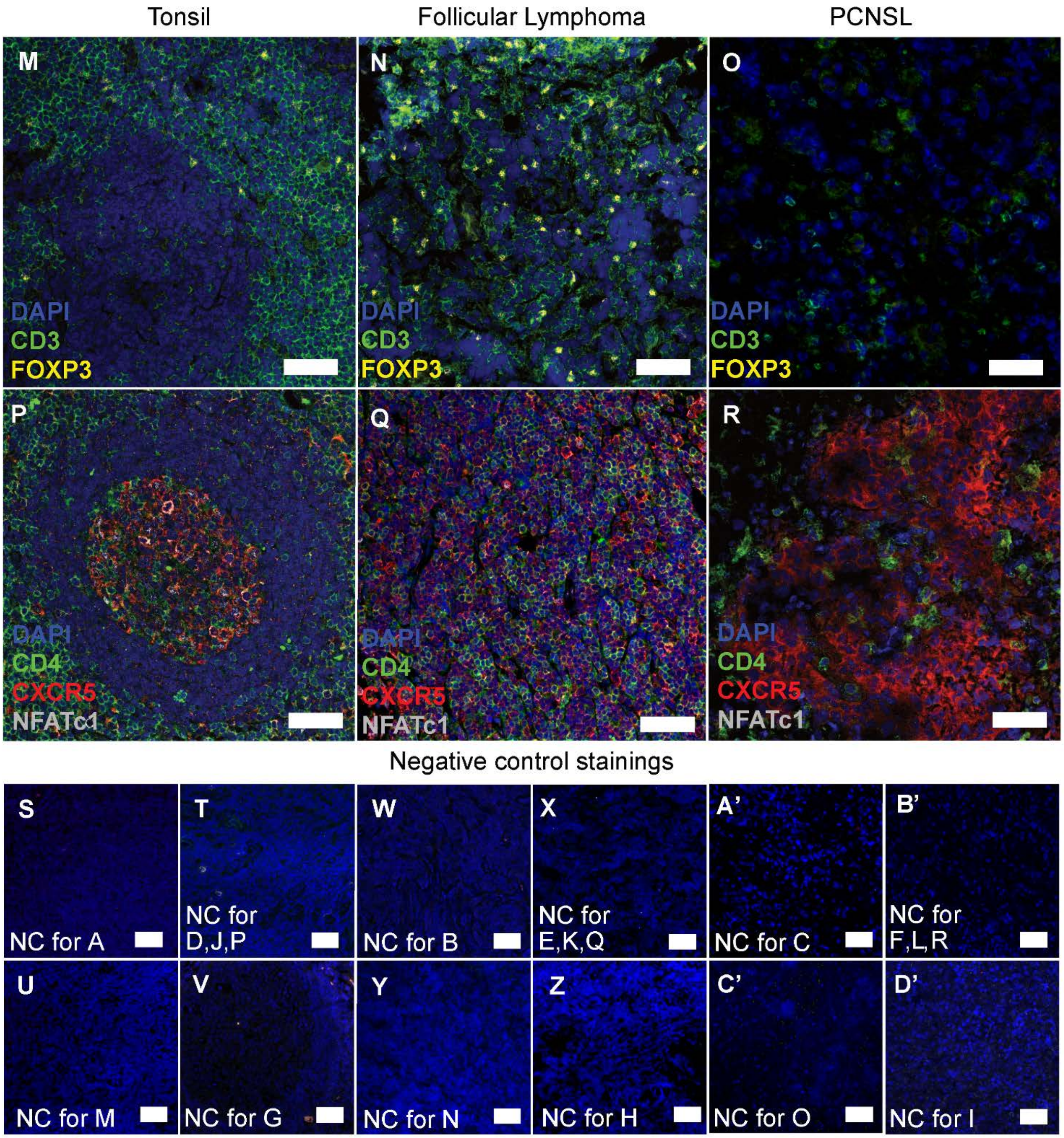

Supplementary Figure 3. IF control stainings of tonsil, follicular lymphoma and primary CNS lymphoma (PCNSL). FFPE sections of tonsils (**A, D, G J, M, P**), follicular lymphoma (**B, E, H, K, N, Q**) and PCNSL (**C, F, I, L, O, R**) were stained for CD3 and CD20 (**A-C**), CD4, CD138 and CD69 (**D-F**), CD3, CD4 and PD-1 (**G-I**), CD3, CD8 and CD27 (**J-L**), CD3 and FOXP3 (**M-O**), or CD4, CXCR5 and NFATc1 (**P-R**). Negative controls were stained with secondary antibody only in tonsils (**S-V**), follicular lymphoma (**W-Z**), PCNSL **(A'-D')**. NC, negative control. Scale bars indicate 100 µm.
